# Regulation of Heparin-Binding EGF-Like Growth Factor by miR-212 and Acquired Cetuximab-Resistance in Head and Neck Squamous Cell Carcinoma

**DOI:** 10.1371/journal.pone.0012702

**Published:** 2010-09-13

**Authors:** Hiromitsu Hatakeyama, Haixia Cheng, Pamela Wirth, Ashley Counsell, Samuel R. Marcrom, Carey Burton Wood, Paula R. Pohlmann, Jill Gilbert, Barbara Murphy, Wendell G. Yarbrough, Deric L. Wheeler, Paul M. Harari, Yan Guo, Yu Shyr, Robbert J. Slebos, Christine H. Chung

**Affiliations:** 1 Division of Hematology/Oncology, Department of Medicine, Vanderbilt University School of Medicine, Nashville, Tennessee, United States of America; 2 Department of Oncology, Johns Hopkins University School of Medicine, Baltimore, Maryland, United States of America; 3 Department of Otolaryngology, Vanderbilt University School of Medicine, Nashville, Tennessee, United States of America; 4 Department of Cancer Biology, Vanderbilt University School of Medicine, Nashville, Tennessee, United States of America; 5 Department of Human Oncology, University of Wisconsin School of Medicine and Public Health, Madison, Wisconsin, United States of America; 6 Department of Biostatistics, Vanderbilt-Ingram Cancer Center, Vanderbilt University School of Medicine, Nashville, Tennessee, United States of America; National Cancer Institute, United States of America

## Abstract

**Background:**

We hypothesized that chronic inhibition of epidermal growth factor receptor (EGFR) by cetuximab, a monoclonal anti-EGFR antibody, induces up-regulation of its ligands resulting in resistance and that microRNAs (miRs) play an important role in the ligand regulation in head and neck squamous cell carcinoma (HNSCC).

**Methodology/Principal Findings:**

Genome-wide changes in gene and miR expression were determined in cetuximab-sensitive cell line, SCC1, and its resistant derivative 1Cc8 using DNA microarrays and RT-PCR. The effects of differentially expressed EGFR ligands and miRs were examined by MTS, colony formation, ELISA, and western blot assays. Heparin-binding EGF-like growth factor (HB-EGF) and its regulator, miR-212, were differentially expressed with statistical significance when SCC1 and 1Cc8 were compared for gene and miR expression. Stimulation with HB-EGF induced cetuximab resistance in sensitive cell lines. Inhibition of HB-EGF and the addition of miR-212 mimic induced cetuximab sensitivity in resistant cell lines. MicroRNA-212 and HB-EGF expression were inversely correlated in an additional 33 HNSCC and keratinocyte cell lines. Six tumors and 46 plasma samples from HNSCC patients were examined for HB-EGF levels. HB-EGF plasma levels were lower in newly diagnosed HNSCC patients when compared to patients with recurrent disease.

**Conclusions/Significance:**

Increased expression of HB-EGF due to down-regulation of miR-212 is a possible mechanism of cetuximab resistance. The combination of EGFR ligand inhibitors or miR modulators with cetuximab may improve the clinical outcome of cetuximab therapy in HNSCC.

## Introduction

Epidermal growth factor receptor (EGFR) is a type 1 membrane tyrosine kinase that plays important roles in differentiation, proliferation, and metastasis of many human cancers, mostly of epithelial origin [1]. EGFR represents one of the four members of the HER family of receptor tyrosine kinases that, upon activation, engage in complex dimerization patterns depending on the repertoire of HER family members expressed by individual cell types. In addition, EGFR has several ligands, including epidermal growth factor (EGF), transforming growth factor-alpha (TGFA), heparin-binding EGF-like growth factor (HB-EGF), amphiregulin (AREG), betacellulin (BTC), epiregulin (EPR) and epigen (reviewed in [2]). These ligands share a consensus sequence, known as the EGF motif, which is important for binding to EGFR. They are frequently produced as transmembrane precursor proteins that require cleavage by cell surface proteases into soluble ligands to bind EGFR. TGFA, HB-EGF, AREG and EPR are cleaved by TNFα-converting enzyme/disintegrin and metalloproteinase 17 (TACE/ADAM17), while EGF is cleaved by ADAM10. Once EGFR is activated, it sets off a cascade of downstream regulator activation including MAPK, AKT and STAT3 (reviewed in [1]).

MicroRNAs (miRs) are single-strand RNAs that regulate mRNA expression [3]. They are transcribed as ∼80-nt long RNA hairpins (primary miRs) and cleaved to ∼60-nt precursor miRs by the protein Drosha in the nucleus [4]. Precursor miRs are transported to the cytoplasm by Exportin 5, further processed to ∼22-nt miRs by the protein Dicer and then loaded into the RNA-induced silencing complex (RISC) to form mature miRs [5], [6]. These mature miRs can inhibit gene transcription by interacting with promoters, as well as induce mRNA degradation or inhibit mRNA translation by forming double-strand RNAs [7], [8]. The interactions among the HER family receptors, receptor ligands and their regulatory miRs are not clearly understood.

Overexpression of EGFR and its ligand, TGFA, is associated with poor prognosis in HNSCC [9]. In line with these data, such EGFR-targeted agents as the small molecule tyrosine kinase inhibitors (i.e. gefitinib and erlotinib) and the monoclonal antibodies (i.e. cetuximab and panitumumab) provide clinical benefit to HNSCC patients [10], [11], [12], [13]. Among these agents, cetuximab is approved by the U.S. Food and Drug Administration for use in HNSCC patients as a monotherapy, as well as in combination with radiation or chemotherapy. Recently several molecular abnormalities were reported to associate with sensitivity or resistance to EGFR inhibitors, including somatic mutations in the EGFR tyrosine kinase domain, *EGFR* gene amplification, *KRAS* mutation, and *MET* amplification [14], [15], [16], [17]. However, these molecular alterations are extremely rare or not significant for predicting response to EGFR inhibitors in HNSCC [18], [19], [20]. Furthermore, most of the patients who are treated with cetuximab develop resistance over time after an initial response, and understanding the mechanism of resistance will be paramount to further optimize the clinical outcome in HNSCC. In this study, we examined mRNA and miR expression levels in a model system for cetuximab resistance to determine possible mechanisms of acquired resistance and to demonstrate that HB-EGF and its regulator miR212 are involved.

## Methods

### Cell lines and materials

Cetuximab-resistant cell line, 1Cc8, was derived from cetuximab-sensitive SCC1 cells as previously described [21]. The culture conditions and sources of 34 HNSCC and a spontaneously immortalized keratinocyte (HaCaT) cell lines used in this study are described in Table S1. Each cell line was authenticated using a short tandem repeat analysis kit, Identifiler (Applied Biosystems, Foster City, CA), as directed at the Johns Hopkins Genetic Resources Core Facility. Cetuximab (Bristol-Myers Squibb, Princeton, NJ) was purchased from the Vanderbilt Pharmacy. Gefitinib was purchased from Tocris Bioscience (Ellisville, MO). TGFA, HB-EGF and AREG were purchased from R&D Systems (Minneapolis, MN). TAPI-2 was purchased from Calbiochem (Los Angeles, CA).

### Ethics Statement

Mouse xenograft studies were performed under an Institutional Animal Care and Use Committee at Office of Animal Welfare Assurance (IACUC OAWA)-approved protocol. The IACUC OAWA specifically approved this study (protocol M/07/351). Frozen tumors and plasma samples from patients with HNSCC were collected with written informed consents under an Institutional Review Board (IRB) Health Sciences Committee (HSC)-approved protocol with “Full Review”, and the experiments specific to this study were conducted under an IRB HSC-approved protocol with “Expedited Review” exempted from obtaining informed consents for being a minimal-risk study at Vanderbilt University Medical Center. The IRB HSC specifically approved this study.

### MTS assay and colony formation assay

For MTS assays, cells were seeded in flat-bottom 96-well culture plates with 1×10^3^ cells per well in quadruplicate at each dose level on day zero. The drugs were added on day one as previously described [21], and the ligands were added every 24 hours. The culture media and cetuximab were changed on day four and growth inhibition was measured on day seven. Gefitinib treated cells were measured on day three. Growth inhibition was measured using the CellTiter 96 Aqueous One Solution Reagent (Promega, Madison, WI) according to the manufacturer's recommendations. Zero dose-treated cells were measured in four independent wells for each cell line and data were expressed as a percentage of growth relative to the zero dose-treated cells. For colony formation assays, cells were seeded with 2×10^4^ cells on Matrigel in flat-bottom 8-well glass plates in triplicate for each condition on day zero. Quantification of colony number and size was performed on day five by image analysis. Representative images were captured by microscope digital camera and analyzed using NIH supplied Image-J software.

### Flow Cytometry

Cells were harvested by trypsinization, washed with phosphate-buffered saline (PBS), and incubated for 30 minutes on ice with FITC-labeled cetuximab (5 µg/ml; Pierce Labeling Kit) or with isotype-matched nonbinding antibody FITC-rituximab (5 µg/ ml) in PBS containing 0.5% BSA and 2 mM EDTA (FACS buffer). After washing with PBS, cells were diluted in 0.5 ml of FACS buffer. Flow cytometry of FITC-labeled cells was performed using a FACS/Calibur Flow Cytometer (Becton Dickinson, Mansfield, MA).

### Total RNA isolation and analyses

Total RNA was isolated from the cell lines and frozen HNSCC tumors using Qiagen RNeasy Mini kit according to the manufacturer's recommendations (Qiagen, Valencia, CA). Frozen HNSCC tumors were examined for tumor cellularity and macro-dissected to enrich for cancer cells to ≥70% before lysis. The quality and quantity of the RNA was determined using the Agilent RNA 6000 NanoLabChip kit and Agilent 2100 bioanalyzer (Agilent Technologies, Santa Clara, CA). For DNA microarray analyses, the RNA was labeled with GeneChip^R^ One-Cycle Target Labeling and Control Reagents and loaded on to the Affymetrix Human Genome U133 plus 2.0 GeneChip according to the manufacturer's recommendations (Affymetrix, Santa Clara, CA). The primary microarray data was normalized using Perfect Match software for further statistical analyses. Normalized microarray data were imported to GeneSpring 10 (Silicon Genetics, Redwood City, CA) and analyzed. Genes that were differentially expressed between cetuximab sensitive and resistant cell lines were selected using one-way ANOVA with FDR<1%. For RT-PCR analyses of EGFR ligands in HNSCC tumors, Applied Biosystems Taqman FAM labeled probes for TGFA, HB-EGF, NRG1, AREG and EGF were obtained and analyzed according to the manufacturer's recommendations (Applied Biosystems, Foster City, CA). All data are MIAME compliant and the raw data have been deposited in Gene Expression Omnibus (GSE21483).

### MicroRNA TLDA assay

Total RNAs were isolated from each cell line using Recover All Total Nucleic Acid Isolation kit according to the manufacturer's recommendations (Ambion INC, Austin, TX). The quality and quantity of the RNA was determined using the Agilent RNA 6000 NanoLabChip kit and Agilent 2100 bioanalyzer (Agilent Technologies, Santa Clara, CA). Total RNA was used to run the ABI Megaplex protocol without pre-amplification (Applied Biosystems, Foster City, CA). A reaction was run using the ABI miRNA Reverse transcription kit (#4366596) and Megaplex RT Human Pool A primers (#4399966). The cDNA per sample was then transferred to a new tube, diluted with water and Taqman Universal PCR Master Mix without UNG, 2X (#4324018). Each sample was then loaded onto its own TLDA card. TLDAs were queued into the ABI7900HT real time PCR machine and run with the ABI default TLDA protocol. Data were normalized based on the control on the TLDA card and analyzed.

### Immunoblotting

Cells were lysed with RIPA lysis buffer [1 mM NaVO3, 1 mM DTT, 1 mM PMSF, phosphatase inhibitor cocktail, and protease inhibitor cocktail mini tablet (Roche, Indianapolis, IN)] and sonicated. Protein concentration was quantified with a standard Bradford absorbance assay. Protein from each sample was fractionated by SDS-PAGE. Proteins were transferred to nitrocellulose membrane and incubated with the appropriate primary antibodies (total and p-EGFR, -HER2, -HER3, -HER4, -FGFR, -MET, -AKT, -MAPK and –STAT3, Smad2, and β-Actin; Cell Signaling Technology, Boston, MA) followed by secondary antibodies. Signal intensity was determined by Imagegauge version 4.1 (Fujifilm, Japan). Each gel was normalized to β-Actin.

### Detection of ligand levels in the conditioned media and in plasma

Cells (4×10^5^ cells/ml per well) were seeded in 6-well culture plates and each condition was tested in triplicate. Sixteen hours later the culture medium was aspirated, the cells were washed with PBS and incubated in serum-free media for 24 hours. Fresh serum-free culture medium containing cetuximab at the specified concentrations was added to each well and the cells were incubated for an additional 24 hours. Cells were isolated by centrifugation and frozen at −80 degree C until assayed. The plasma samples from patients were processed and stored according to the standardized institutional protocol until analysis. All ligands levels were quantified by sandwich ELISA assays using Duoset ELISA Development System (R&D systems, Minneapolis, MN) according to the manufacturer's recommendations.

### Modulation of HB-EGF and miR-212 expression

Human GIPZ lentiviral shRNAmir (# RHS4430-98481014) individual clone used to silence HB-EGF expression and a GIPZ lentiviral negative control vector were purchased from Open Biosystems (Rockford, IL). Infectious viruses were produced by co-transfecting the lentiviral vector and packaging constructs into 293FT cells (Invitrogen, Carlsbad, CA) using Lipofectamine 2000 (Invitrogen, Carlsbad, CA). Infectious lentivirus particles were harvested 48 hours after transfection. 1Cc8 cells were infected with each virus and then cultured for 5 days. The miRNA mimics/inhibitor and negative controls were purchased from Dharmacon (Lafayette, CO) and introduced into cells by Lipofectamine 2000. Cells were collected after 48 hours post-transfection. Binding of miR-212 to HB-EGF was determined by co-transfecting 50 nM of miR-212 mimics (Dharmacon, Lafayette, CO) and 1 µM of miRNA 3′UTR target expression clone for Human NM_0019445.1-HB-EGF (GeneCopoeia Inc., Rockville, MD) into SCC1 and 1Cc8 cells with lipofectamine 2000 (Invitrogen, Carlsbad, CA) in 6-well plate. Firefly and Renilla luciferase activities were measured 48 hours after transfection using Luc-Pair miR Luciferase Assay Kit (GeneCopoeia Inc., Rockville, MD) according to the manufacturer's recommendations.

### Cetuximab treatment of cell line xenografts *in vivo*

Athymic nude mice (4 to 6-week-old females) were obtained from Harlan Laboratories (Indianapolis, IN). A suspension of 4×10^6^ cells from each cell line in HBSS was injected subcutaneously into the right flank of mice. Tumor volumes were measured in length and width twice a week. Tumor volumes were calculated using the formula (length x width^2^xπ)/6. All planted tumors were grown for 6 to 8 days until average tumor volume reached 30 mm^3^ before treatment. Five mice per group were treated with intraperitoneal injections of 50 mg/kg cetuximab weekly for 4 weeks. All mice were sacrificed 28 days after the first treatment.

## Results

### Characterization of EGFR inhibitor sensitivity in HNSCC cell lines

**Figure 1:**
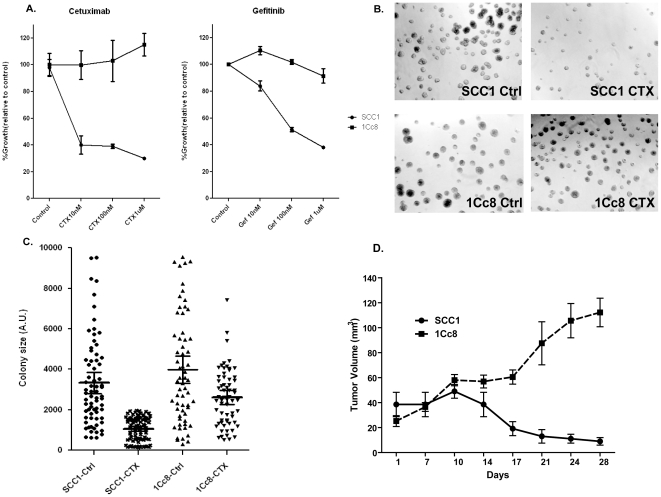
Determination of cetuximab sensitivity. Cetuximab response determination of SCC1 and 1Cc8 HNSCC cell lines by; A) MTS assay, B) colony formation assay, C) graphical presentation of the colony formation assay, A. U.- Arbitrary Unit, and D) growth inhibition in mouse xenografts. CTX-cetuximab. Ctrl-control.

To assess sensitivity to EGFR inhibitors, MTS assays were performed with SCC1 and 1Cc8 cells after treatment with cetuximab and gefitinib (Figure 1A). While SCC1 showed high sensitivity to cetuximab (IC_50_, 8.0 nM) and gefitinib (IC_50_, 273 nM), 1Cc8 showed resistance to cetuximab (IC_50_, >1 µM) and gefitinib (IC_50_, >1 µM). As gefitinib is currently not relevant in the treatment of HNSCC patients, we focused subsequent studies on cetuximab resistance. We chose 100 nM (15 µg/ml) dose of cetuximab for additional studies because this is a receptor saturating concentration optimal to inhibit growth of EGFR-dependent cancer cells in culture [22] and achieved at steady-state in the plasma of patients receiving cetuximab [23]. Further, there was no difference in cell viability or proliferation observed beyond 100 nM in our cell lines (data not shown). Colony formation assays and *in vivo* mouse xenograft studies confirmed our initial MTS assay results (Figure 1B–D).

### Increased EGFR ligand expression is associated with cetuximab resistance

**Figure 2:**
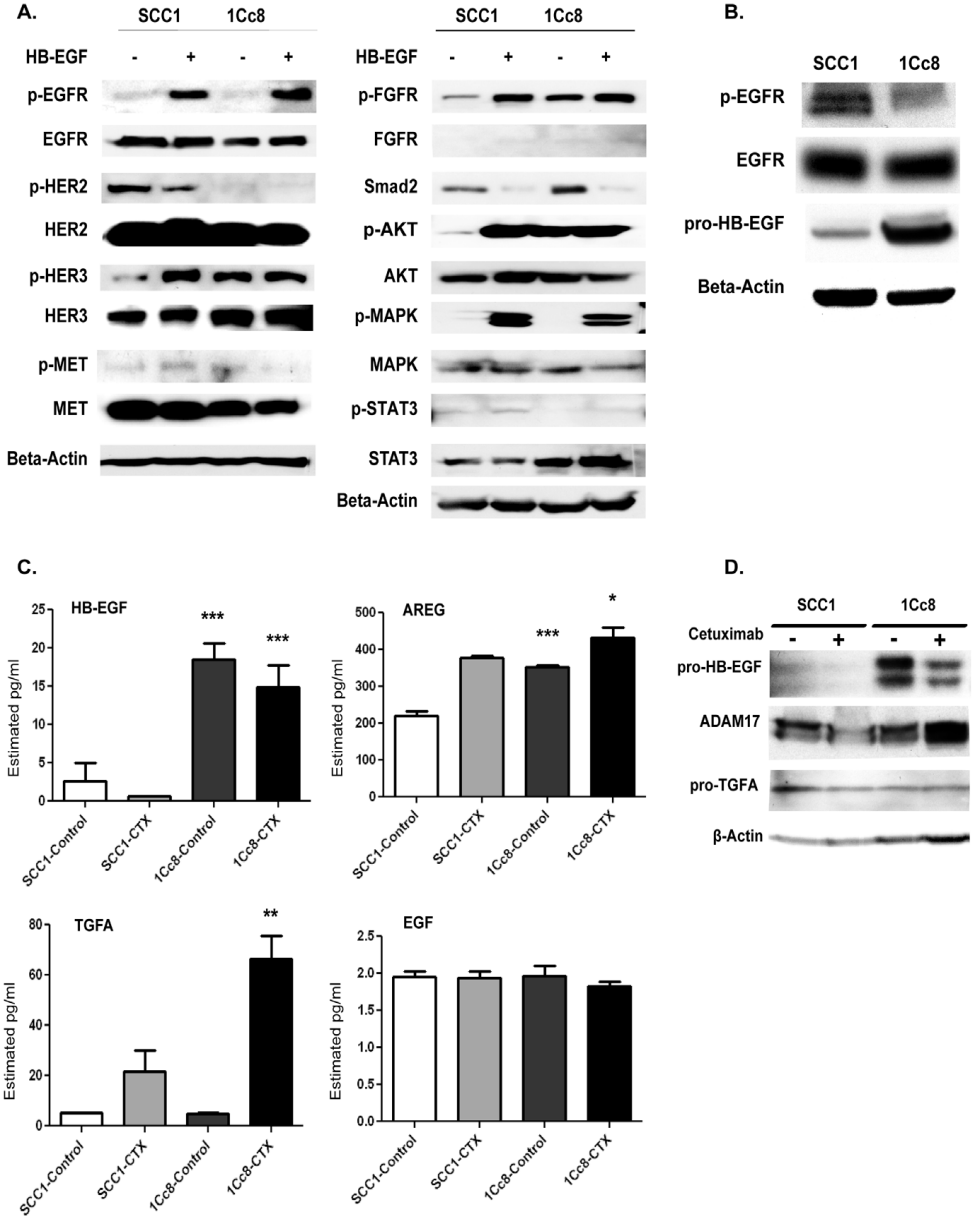
Determination of the EGFR ligand expression and receptor kinase activation in SCC1 and 1Cc8 cell lines. A) Western blot analyses of receptor tyrosine kinases and their downstream proteins with/without HB-EGF stimulation. B) EGFR and pro-HB-EGF protein expression levels in mouse-xenograft tumors generated from SCC1 and 1Cc8. C) EGFR ligand levels in conditioned media of SCC1 and 1Cc8 with/without cetuximab treatment determined by ELISA assays. P-values were generated comparing SCC1 versus 1Cc8 for control or cetuximab treatment (* P<0.05, ** P<0.01, ***P<0.001). D) Western blot analyses for pro-HB-EGF, pro-TGFA and TACE/ADAM17 levels with/without cetuximab treatment in cell lysates from SCC1 and 1Cc8. AREG- amphiregulin, EGF- epidermal growth factor, HB-EGF- heparin-binding EGF-like growth factor, TGFA- transforming growth factor alpha.

The EGFR expression levels and the ability of cetuximab to bind EGFR were examined using flow cytometry (Figure S1A). There was no apparent difference in cetuximab binding to EGFR; however, 1Cc8 had lower expression of EGFR compared to SCC1 (log fluorescence intensity of 34.8 in 1Cc8 cells versus 94.4 of SCC1 cells). The EGFR mRNA and protein expression levels *in vitro* and in tumor lysates from mouse xenografts were lower in 1Cc8 when compared to SCC1 (Figure S1B and Figure 2A–B). As previously reported [21], the phospho-AKT was significantly higher in 1Cc8 when compared to SCC1 and there was increased activation of HER3.

To further detect differentially expressed genes and pathways that are associated with increased AKT activation despite decreased EGFR levels in cetuximab resistant cells, we performed supervised analyses comparing SCC1 and 1Cc8 (the microarray data were deposited at Gene Expression Omnibus, GSE21483). We identified 900 probes with greater than two-fold expression difference and with t-test p-value of less than 0.01 (Table S2). Using these probes, Ingenuity Pathway Analysis was performed examining the EGFR signaling pathway and its related genes. Among these genes, only HB-EGF and PIK3R3 were up-regulated in 1Cc8 (Figure S1C). To confirm these data, we examined soluble protein expression levels of HB-EGF and three other EGFR ligands, AREG, TGFA and EGF for relative comparison with HB-EGF in conditioned culture media and cell lysate before and after treatment with cetuximab. Conditioned culture media from 1Cc8 demonstrated higher levels of active soluble HB-EGF and AREG compared to SCC1 (Figure 2C). TGFA levels were not significantly different between the cell lines before treatment, but cetuximab treatment resulted in a dramatic increase in TGFA levels in 1Cc8 compared to SCC1. EGF levels were extremely low in the two cell lines and did not vary with cetuximab treatment. In the cell lysates, pro-HB-EGF levels were significantly higher in 1Cc8 compared to SCC1; however, pro-TGFA levels did not differ between the two cell lines (Figure 2D). In mouse xenograft tumors, the pro-HB-EGF level was also higher in 1Cc8 when compared to SCC1 (Figure 2B). Western blot analysis of TACE/ADAM17 showed that cetuximab treatment increased TACE/ADAM17 expression in 1Cc8 cells, but not in SCC1 cells (Figure 2D). These data suggest that HB-EGF may not depend on its protease for activation while a rapid increase in soluble TGFA level is caused by an increased activity of its protease [24], [25].

### Increased expression of HB-EGF and receptor kinase crosstalk

Further evaluation of the stimulatory effects of HB-EGF on downstream proteins showed that HB-EGF robustly activated AKT, MAPK and STAT3 (Figure 2A). With recent evidence that HB-EGF can induce epithelial-to-mesenchymal transition (EMT) [26], we also examined expression of fibroblast growth factor receptor (FGFR) and Smad2, a downstream effector of transforming growth factor-beta receptor. Interestingly, phospho-FGFR and Smad2 levels were higher in 1Cc8 when compared to SCC1, and HB-EGF stimulated FGFR activation and decreased expression of Smad2 (Figure 2A).

**Figure 3:**
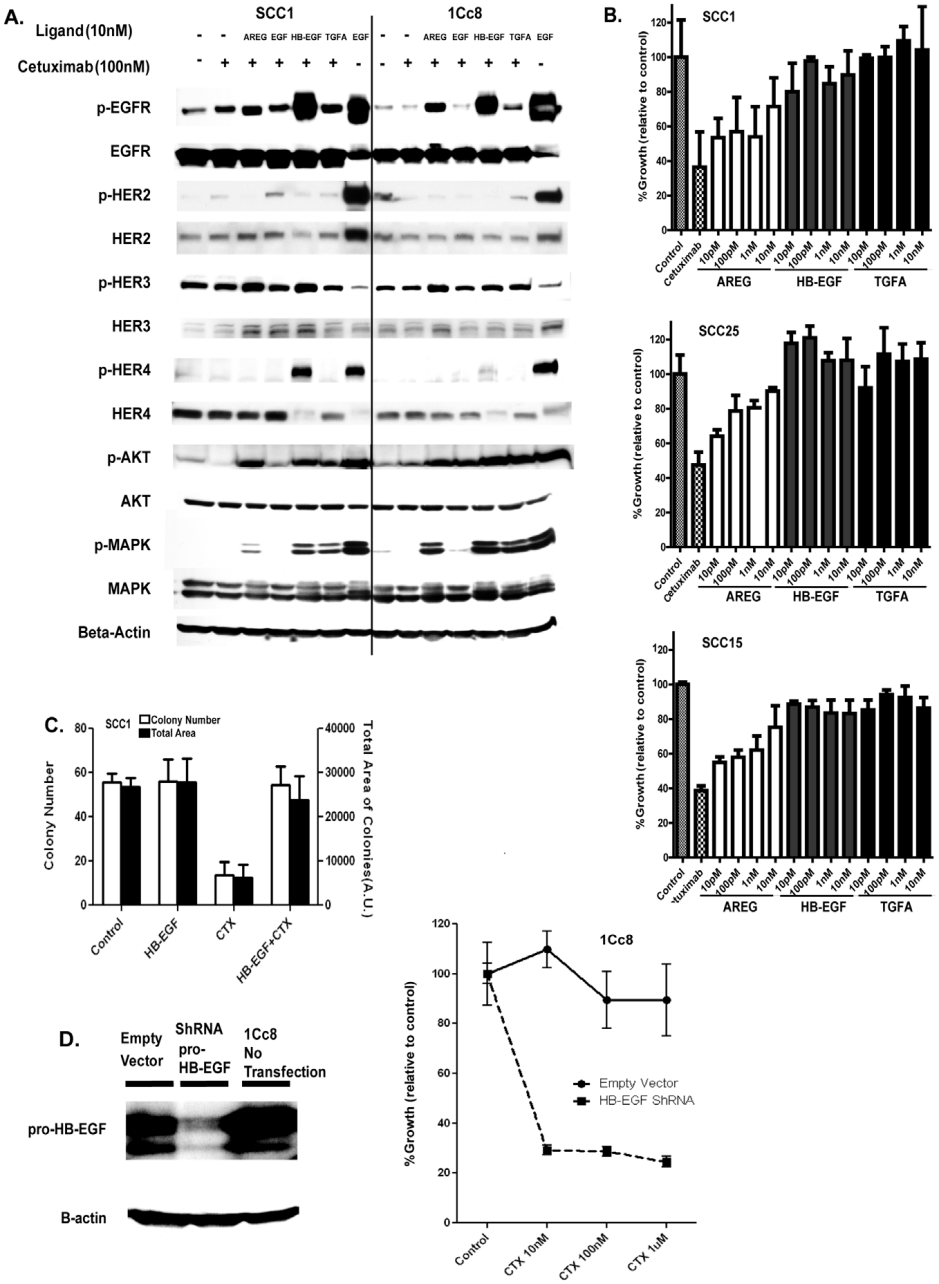
Effects of EGFR ligands in cetuximab sensitivity. A) Western blot analyses of HER family receptor kinases and their downstream proteins with/without EGFR ligand stimulation in the presence of cetuximab. B) Induction of cetuximab resistance by exogenous EGFR ligands in three cetuximab sensitive HNSCC cell lines determined by MTS assay. C) Induction of cetuximab resistance by exogenous HB-EGF in SCC1 determined by colony formation assay. D) Pro-HB-EGF level in the cell lysate of 1Cc8 after knockdown of HB-EGF shown in Western blot. Growth inhibition rate of 1Cc8 cells transfected with shRNA HB-EGF and an empty vector measured by MTS assay.

With the evidence of differential activation of HER family receptors, the ability to activate each receptor for each ligand was examined in the presence of cetuximab (Figure 3A). Three ligands, AREG, HB-EGF and TGFA, activated EGFR even in the presence of cetuximab in both cell lines, but the effects of EGF on EGFR in the presence of cetuximab were minimal. The EGFR activation was the highest after stimulation with HB-EGF, and only HB-EGF could activate HER-4 with lesser degrees in 1Cc8 when compared to SCC1, consistent with the lower expression of the receptors in 1Cc8. However, phospho-AKT and –MAPK levels were higher in 1Cc8 further supporting our finding that there may be activation of receptors other than EGFR.

### HB-EGF knockdown reverses resistance to cetuximab

To establish a causal relationship between increased EGFR ligand levels and cetuximab resistance, we repeated cell proliferation assays in the presence of exogenous ligands (AREG, HB-EGF and TGFA) in the cetuximab sensitive cell line, SCC1 (Figure 3B). Addition of ligand to culture media induced cetuximab resistance in SCC1. Low concentrations of TGFA and HB-EGF were sufficient to confer resistance to cetuximab, whereas addition of AREG at these concentrations was not as effective. It is possible that TGFA and HB-EGF may have a higher affinity to the receptor compared to AREG; therefore, TGFA and HB-EGF may more readily compete for receptor binding in the setting of prolonged cetuximab exposure. These findings were confirmed in two additional cetuximab-sensitive HNSCC cell lines (SCC25, IC_50_ = 6.19 nM and SCC15, IC_50_ = 6.45 nM, Figure 3B). The effects of HB-EGF on cetuximab resistance were further examined by colony formation assays in serum-free media. As seen in the MTS assay, addition of HB-EGF effectively reversed cell growth inhibition by cetuximab in SCC1 (Figure 3C).

To further investigate the role of increased TGFA after cetuximab treatment in cetuximab-resistant 1Cc8 cells, we examined the effect of TACE/ADAM17 inhibition on cell growth/viability by treating the cells with TNF protease inhibitor-2 (TAPI-2), a broad-spectrum inhibitor of MMPs and TACE/ADAM17 [27]. Results from the MTS assay show TAPI-2 as a monotherapy has a limited effect on SCC1 and 1Cc8 (SCC1, IC_50_ = 30.32 µM, and 1Cc8, IC_50_>100 µM). In combination with cetuximab, TAPI-2 enhanced the effect of cetuximab in SCC1, but cellular growth rates were similar to TAPI-2 monotherapy in 1Cc8 (Figure S2A). Therefore, it appears that increased TGFA levels after cetuximab treatment do not significantly contribute to cetuximab resistance seen in 1Cc8. The elevated TGFA level may simply reflect inhibition of TGFA binding to EGFR by cetuximab and subsequent decrease in the TGFA/EGFR internalization. Based on these data, we further investigated the significance of HB-EGF in cetuximab resistance by using HB-EGF-specific shRNA to silence its expression. Transfected 1Cc8 cells expressed lower levels of pro-HB-EGF accompanied by increased sensitivity to cetuximab (Figure 3D).

### HB-EGF is regulated by miR-212 and decreased expression of miR-212 is associated with cetuximab resistance

**Figure 4:**
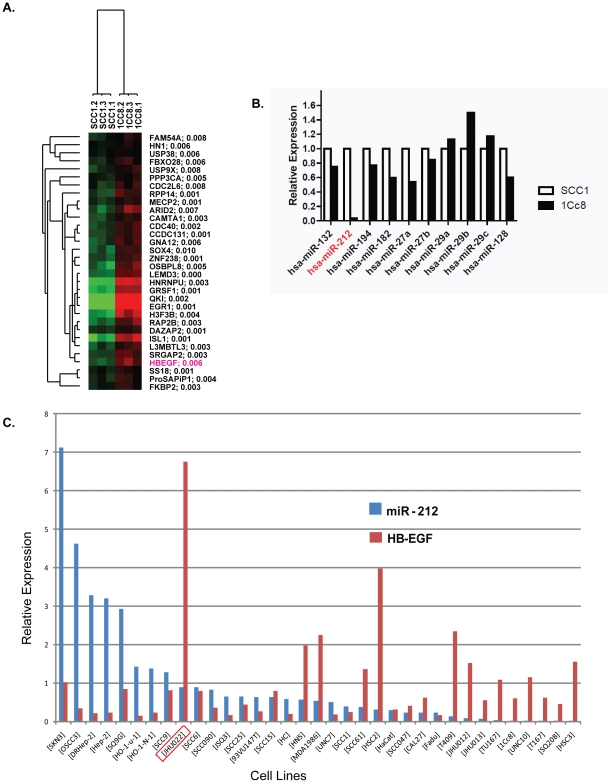
Identification of microRNA-212 as a potential regulator of HB-EGF expression. A) Top 20 genes putatively targeted by miR-212. The numbers next to the gene symbols are p-values. Red: higher gene expression, Green: lower gene expression. B) Relative expression of top 10 miRs targeting HB-EGF in SCC1 and 1Cc8. C) Relative expression levels of miR-212 and HB-EGF in 34 HNSCC cell lines and a keratinocyte cell line. HB-EGF expression data were obtained from DNA microarray analyses and miR-212 expression data were obtained from TLDA microRNA arrays.

Because regulation of EGFR ligand levels appears to be a dynamic process, we examined the role of miRs in the ligand regulation, which is a rapid mechanism of regulating the mRNA expression levels, and its association with cetuximab sensitivity. We performed miR expression analyses using RT-PCR-based arrays examining 384 unique miRs. Among the differentially expressed miRs, miR-212 showed a 27-fold decrease in 1Cc8 relative to SCC1 (Table 1). Because a miR can regulate multiple genes, we obtained a list of 205 genes in Targetscan 5.1 (http://www.targetscan.org) that are putatively targeted by miR-212, and examined their gene expression levels in SCC1 and 1Cc8. There were 32 genes that were differentially expressed between SCC1 and 1Cc8 with p-values of less than 0.05 by t-test (Figure 4A). Among the 32 genes, HB-EGF was the only gene known to have direct binding with EGFR. Since a single gene can be regulated by many miRs, we obtained a list of 10 miRs in Targetscan 5.1 (http://www.targetscan.org) that putatively target HB-EGF and found that only miR-212 showed a significant difference between SCC1 and 1Cc8 (Figure 4B). To determine whether this association could be generalized to other cell lines, we examined HB-EGF and miR-212 expression levels in an additional 32 HNSCC cell lines and a keratinocyte cell line (Figure 4C). In this analysis, there was an inverse correlation between HB-EGF and miR-212 levels (Spearman r = −0.37, p = 0.036), with the exception of JHU022. Interestingly, JHU022 was the only cell line with a heterozygous deletion of the region containing miR-212 in chromosome 17p13 using available SNP data (Figure S2B).

**Table 1:** Top 20 differentially expressed microRNAs.

SCC1>1Cc8		SCC1<1Cc8	
miRNA	Fold Change	miRNA	Fold Change
hsa-miR-212-4373087	27.8	hsa-miR-146a-4373132	41.4
hsa-miR-423-5p-4395451	9.5	hsa-miR-93-4373302	13.0
hsa-miR-483-5p-4395449	7.0	hsa-miR-202-4395474	11.1
hsa-miR-628-5p-4395544	7.0	hsa-miR-597-4380960	7.3
hsa-miR-361-5p-4373035	5.1	hsa-miR-523-4395497	5.7
hsa-miR-95-4373011	5.0	hsa-miR-138-4395395	5.7
hsa-miR-342-3p-4395371	4.5	hsa-miR-135a-4373140	3.6
hsa-miR-219-1-3p-4395206	4.3	hsa-miR-886-3p-4395305	3.6
hsa-miR-491-5p-4381053	4.0	hsa-miR-542-3p-4378101	3.4
hsa-miR-375-4373027	4.0	hsa-miR-193a-5p-4395392	3.0

SCC1: cetuximab-sensitive cell line.

1Cc8: cetuximab -resistant cell line.

**Figure 5:**
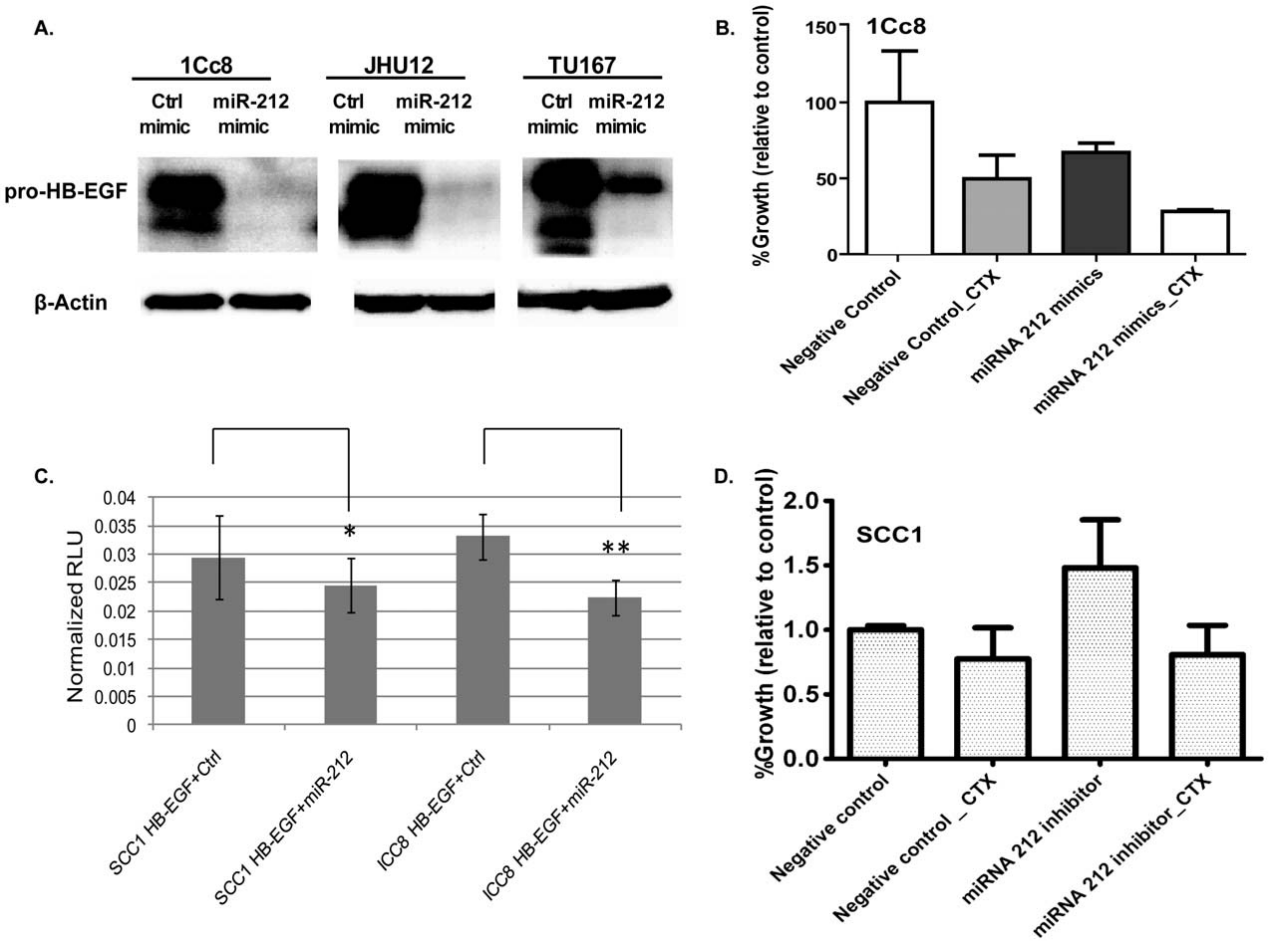
MicroRNA-212 regulates HB-EGF expression in cetuximab resistant cells. A) Pro-HB-EGF expression following the transfection with miR-212 mimics in three cetuximab resistant cell lines. B) Growth inhibition rate of 1Cc8 cells with exogenous miR-212 mimics, cetuximab or a combination of miR-212 mimics and cetuximab measured by colony formation assay. C) MicroRNA-212 directly regulates HB-EGF by binding to the 3′ untranslated region of HB-EGF in cetuximab resistant 1Cc8 cells while it was not significant in cetuximab sensitive SCC1 cells (*, p = 0.47; **, p = 0.038). RLU – Raw Light Units. D) Growth rate comparison of SCC1 cells in the presence of cetuximab, exogenous miR-212 inhibitor, or a combination of miR-212 inhibitor and cetuximab measured by colony formation assay.

We further established a negative regulation of miR-212 on HB-EGF by adding a miR-212 mimic into 1Cc8. The resistant cell line, 1Cc8, was transfected with a miR-212 mimic, or with a negative control. After transfection, HB-EGF expression was dramatically decreased compared to the negative control (Figure 5A). Using two additional cell lines (JHU12 and TU167) in which expression of HB-EGF and miR-212 were similar to that in 1Cc8, we determined that both cell lines showed a clear down-regulation of HB-EGF expression following transfection with the miR-212 mimic. Furthermore, a combination of the miR-212 mimic and cetuximab was more effective in growth inhibition in colony formation assay using 1Cc8 compared to cetuximab or the miR-212 mimic alone (Figure 5B). Direct binding of miR-212 to 3′UTR of HB-EGF was confirmed using a luciferase assay which showed significant decrease of HB-EGF in the presence of miR212 in 1Cc8 (p = 0.038) while the significance was not in SCC1 (p = 0.47, Figure 5C). In addition, we examined the effects of miR-212 antagomir/inhibitor in SCC1 by colony formation assay on matrigel. Inhibition of miR-212 increased growth as expected, but the antagomir did not significantly affect the cetuximab sensitivity in SCC1 (Figure 5D). This suggests that the regulation of HB-EGF by miR-212 may be specific to a biological context of cetuximab resistance in 1Cc8, and HB-EGF may be regulated by mechanisms other than miR-212 in cetuximab sensitive SCC1 cells.

### HB-EGF expression levels vary significantly in tumors and plasma from HNSCC patients taken at the time of diagnosis and of recurrence

**Figure 6:**
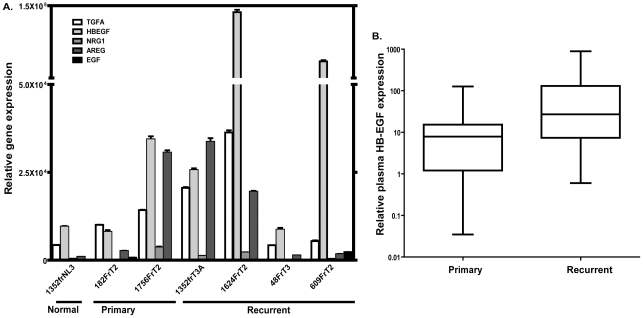
The HB-EGF levels in patients with HNSCC. A) Relative expression levels of five ligands in one normal oral mucosa and six tumors from patients with HNSCC. B) HB-EGF protein levels in plasma from patients with HNSCC. “Primary” plasma samples were taken at the time of diagnosis. “Recurrent” samples were taken at the time of recurrence.

To examine whether there is differential expression of EGFR ligands in human tumors, we determined expression levels of TGFA, HB-EGF, NRG1, AREG and EGF by RT-PCR in one normal oral mucosa, two HNSCC tumors taken at the time of diagnosis, and four HNSCC tumors taken at the time of recurrence (Figure 6A and Table 2). TGFA, HB-EGF and AREG were consistently expressed in all tumors, and the expression levels of HB-EGF were much higher compared to TGFA and AREG in HNSCC. As seen in the HNSCC cell lines, the expression levels of EGF in the tumors were very low. Because the tumors taken at the time of recurrence suggested having higher expression of HB-EGF compared to those taken at the time of diagnosis before any treatment, we examined HB-EGF levels in 16 plasma samples taken at the time of diagnosis and 30 plasma samples taken at the time of recurrence (Figure 6B and Table 3). This analysis revealed that the average plasma HB-EGF level in patients with recurrence was more than five times higher than in patients with newly diagnosed tumors: 95 pg/ml versus 23 pg/ml, respectively (p = 0.017, Wilcoxon rank test). It may have a clinical implication since cetuximab is currently used to treat recurrent disease. Our results suggest that it may be better employed in upfront therapy rather than after recurrence with higher levels of HB-EGF. However, the direct correlation between HB-EGF expression levels and response to cetuximab could not be ascertained due to lack of samples from patients uniformly treated with cetuximab in a sample size with a statistical power.

**Table 2:** Tumor characteristics from patients with head and neck squamous cell carcinoma.

Patient ID	Tumor Type	Age at Dx	Ethnicity	Sex	Tumor Site	TNMStage at Dx	Tumor Diff
182frT2	Primary tumor	59	W	M	OP	T1N3M0	Mod
1756frT2	Primary tumor	45	W	M	L	T4N2cM0	Mod
1352frT3A	Recurrent primary tumor	51	W	F	OC	T1N0M0	Mod
1624frT2	Recurrent primary tumor	62	W	F	OC	T2N0M0	Mod
48frT3	Recurrent primary tumor	55	W	M	HP	T2N1M0	Poor
609frT2	Recurrent lymph node	65	W	M	OP	T3N2cM0	Mod

Dx: diagnosis, OP: oropharynx, L: larynx, OC: oral cavity, HP: hypopharynx, Tumor Diff: histological differentiation by pathology, Mod: moderately differentiated, Poor: poorly differentiated.

**Table 3:** Plasma characteristics from patients with head and neck squamous cell carcinoma.

		Plasma taken at Dx (n = 16)	Plasma taken at recurrence (n = 30)	Total
**Age (mean)**		59	54	N/A
**Sex**	**F**	3	6	9
	**M**	13	23	36
	**N/A**	0	1	1
**Tumor Subsites**	**OC**	8	10	18
	**OP**	7	11	18
	**HP**	0	1	1
	**L**	0	6	6
	**Other**	1	1	2
	**N/A**	0	1	1
**Tumor Stage at Diagnosis**	**1**	0	4	4
	**2**	1	2	3
	**3**	3	5	8
	**4**	12	18	30
	**N/A**	0	1	1
**Tumor differentiation**	**Well**	4	2	6
	**Moderate**	10	15	25
	**Poor**	2	11	13
	**N/A**	0	2	2

## Discussion

Overexpression of EGFR is associated with poor prognosis in HNSCC. The EGFR inhibitor cetuximab is the only molecularly targeted agent to show significant survival benefits in HNSCC patients as monotherapy or in combination with radiation and/or chemotherapy [9], [12], [13]. However, little is known about the mechanisms of cetuximab resistance in HNSCC. In this paper, we demonstrate that increased expression of HB-EGF regulated by miR-212 and activation of receptor kinases other than EGFR by HB-EGF may play an important role in acquired resistance to cetuximab.

We observed increased expression of EGFR ligands and decreased expression of EGFR in cetuximab-resistant cells. In a previous study describing this cell line model of acquired cetuximab resistance, the resistant cells (1Cc8) showed increased activation of EGFR, HER3, and MET and subsequent activation of AKT compared to sensitive cells (SCC1) [21]. In the current study, while we observed increased expression of HER3, MET, and AKT in 1Cc8 cells, we did not observe increased levels of EGFR in both *in vitro* and *in vivo* studies. This partial difference may be due to increased activation of EGFR by up-regulation of ligands causing increased receptor internalization and the lower detectable level of EGFR in the current study. In addition, the current study employed serum-starved cells to isolate the impact of specific EGFR ligand stimulation. In the previous study, experiments were conducted with 10% fetal bovine serum in the culture medium, thus up-regulation of EGFR may be the result of stimulation by other growth factors in the medium.

Epidermal growth factor receptor ligands have been studied in several cancers as potential biomarkers for EGFR-targeted therapy; however, the results have been mixed depending on organ sites and clinical specimens used for testing [16], [18]. In a study by Cohen *et al.*, changes in serum TGFA levels in patients treated with gefitinib was not associated with their clinical response to gefitinib [18]. In a study by Mutsaers *et al.*, TGFA levels were also increased in a dose-dependent manner in the plasma of EGFR-negative colon cancer patients during cetuximab treatment [25]; however, increased TGFA levels did not associate with cetuximab response in a colon cancer clinical trial [28]. These data are supported by our findings that a TACE/ADAM17 inhibitor did not reverse cetuximab resistance in our model cell line. However, increased expression levels of AREG and EPR in tumors of colon cancer patients are associated with cetuximab sensitivity. In contrast, our results suggest that increased HB-EGF may be correlated with cetuximab resistance. This discrepancy could be due to tissue specificity of the EGFR ligand regulation, or expression of other HER family receptors and downstream response upon EGFR activation, which is poorly understood in the context of EGFR inhibitor resistance at this time. In addition, it could be that the association to cetuximab resistance is due to characteristics of HB-EGF itself compared to other ligands.

Heparin-binding EGF-like growth factor is known to bind both EGFR and HER4 and has several unique properties compared to other EGFR ligands (reviewed in [29], [30]). HB-EGF is transcribed as a transmembrane protein (pro-HB-EGF) and cleaved at the juxtamembrane domain into soluble HB-EGF (sHB-EGF), inducing a mitogenic response in keratinocytes [31], [32]; however, unlike other ligands, pro-HB-EGF is also biologically active through juxtacrine signaling to neighboring cells [33]. In our study, both sHB-EGF and pro-HB-EGF levels were elevated in cetuximab-resistant cells. In addition, the carboxy-terminal fragment of pro-HB-EGF (HB-EGF-C) is known to translocate to the nucleus and bind promyelocytic leukemia zinc finger protein (PLZF), which is a transcription factor that negatively regulates the cell cycle through suppressing the expression of cyclin A [34]. Binding of HB-EGF-C to PLZF causes nuclear export of PLZF and induces cell cycle progression [34], [35]. Knockout mice lacking HB-EGF result in perinatal or postnatal lethality from defects in heart chamber and valve formation, abnormal development of lungs, and a significant defect in epidermal wound healing [36], [37] while knockout mice lacking all three major EGFR ligands (EGF, TGFA and AREG) result in mammary gland impairment and small intestine defects but are viable and fertile [38], [39]. In line with its role in keratinocyte migration, there is direct evidence that HB-EGF can induce EMT, enhance metastasis, and modulate chemotherapy resistance [26], [40], [41], [42]. Interestingly, one of the proposed resistance mechanisms that associate with EGFR inhibitors in HNSCC is EMT [43], [44], [45]. In our previous work, we showed that an EMT-linked gene expression profile is associated with a high risk of recurrence in HNSCC [46]. Our data suggest that HB-EGF may have a direct role in cetuximab resistance and EMT potentially by activation of FGFR in the setting of prolonged cetuximab exposure. The characteristics of juxtacrine and paracrine signaling and a mechanism of FGFR activation upon HB-EGF stimulation, as well as its role in EMT in the context of cetuximab resistance are currently being investigated.

Lastly, this is the first study to show that a miR has an important role in regulating a receptor ligand. While the importance of miR regulation in cancer has been known for several years, the regulation of receptor ligands by miRs has not previously been reported. Our data implicate miR-212 as a critical component of HB-EGF regulation in the setting of cetuximab resistance and that its level is inversely correlated with HB-EGF levels in various HNSCC cell lines. In a comprehensive analysis of miRs in HNSCC cell lines by Tran, *et al.*, miR-212 was reported to be one of the miRs with low expression [47]. We also found that JHU022, a cell line with a deletion in the chromosomal region containing miR-212, had an aberrantly high level of HB-EGF. While the extent of oncogenic dependency to a single copy loss of miR-212 and subsequently increased HB-EGF in JHU022 requires further investigation, these data propose a novel mechanism that abnormal regulation of EGFR ligands by genetic gain or loss of miR-containing loci may promote carcinogenesis.

In conclusion, our study suggests that one potential mechanism of acquired resistance to cetuximab involves increased expression of HB-EGF, and that HB-EGF is regulated by miR-212 and may have an active role in inducing EMT. Further studies are required to understand the role of EGFR ligands and their regulation through miRs, and the induction of EMT as a novel approach to overcome EGFR inhibitor resistance.

## Supporting Information

Figure S1 A) Binding of cetuximab to EGFR was analyzed by flow cytometry. Isotype control antibody (rituximab) histogram is indicated as a gray line and cetuximab as a black line. B) The EGFR mRNA expression levels in SCC1 and 1Cc8. C) A network of EGFR pathway-associated genes determined by Ingenuity Pathways Analysis using 900 probes that are differentially expressed between cetuximab-sensitive and -resistant cell lines (SCC1 and 1Cc8). Red: genes that are expressed at higher levels in the cetuximab-resistant cell line compared to the cetuximab-sensitive cell line.
(1.11 MB TIF)

Figure S2 A) Growth inhibition rate of SCC1 and 1Cc8 following treatment with cetuximab, TAPI-2 or a combination of both drugs measured by MTS assay. B) Chromosomal map of 17p13.3 containing miR-212 coding region using genome wide single nucleotide polymorphism data of JHU022.
(1.01 MB TIF)

Table S1 Culture media and sources of 34 head and neck cancer cell lines and HaCaT cells.
(0.05 MB DOC)

Table S2 900 probes that are differentially expressed between SCC1 and 1Cc8.
(0.15 MB PDF)
