# Supplementary figures and images for: Regulation of Heparin-Binding EGF-Like Growth Factor by miR-212 and Acquired Cetuximab-Resistance in Head and Neck Squamous Cell Carcinoma
Source: PLoS One. 2010 Sep 13;5(9):e12702. doi: 10.1371/journal.pone.0012702 (PMC2938338; doi:10.1371/journal.pone.0012702)

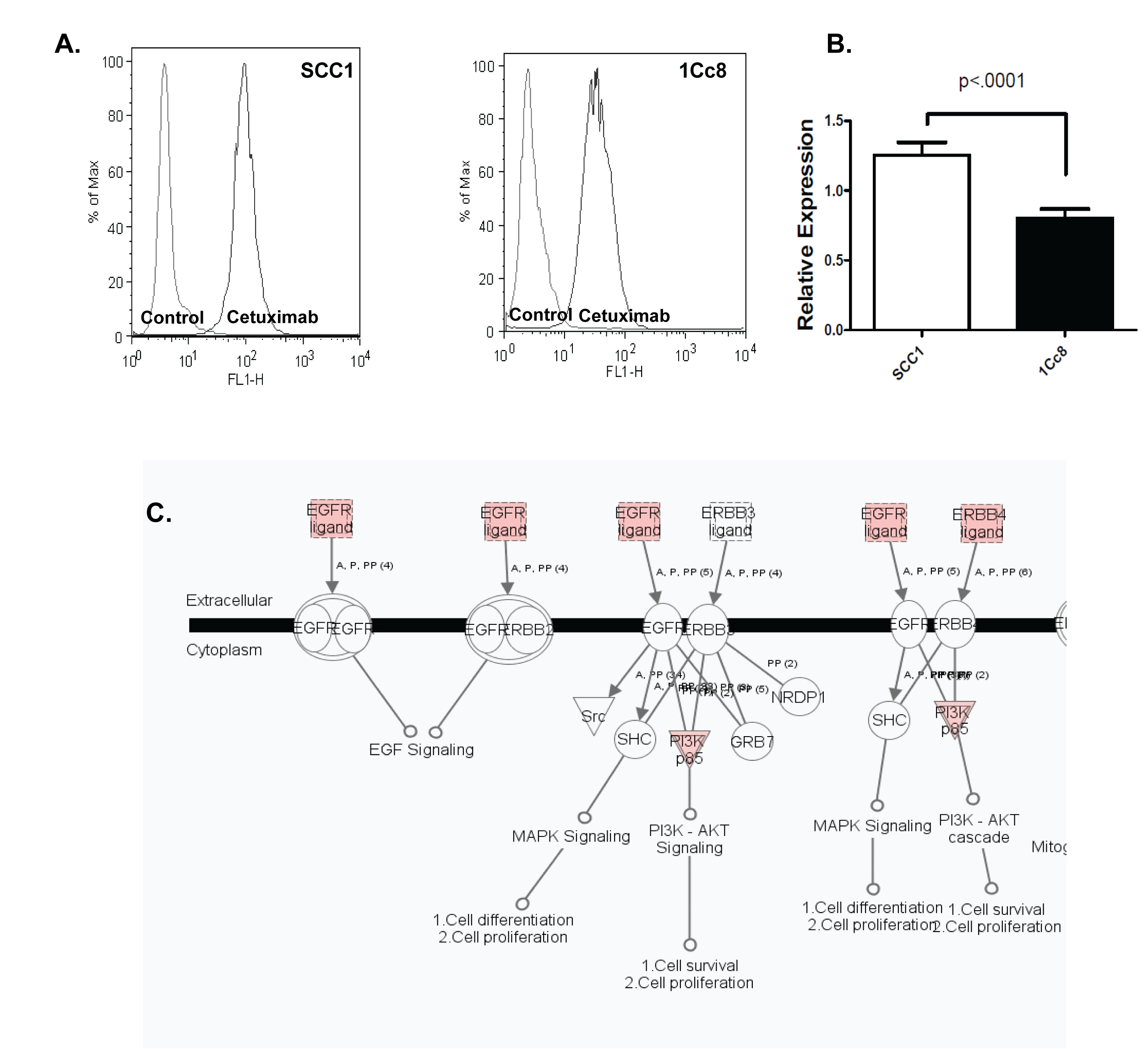

Supplement: Figure S1 — A) Binding of cetuximab to EGFR was analyzed by flow cytometry. Isotype control antibody (rituximab) histogram is indicated as a gray line and cetuximab as a black line. B) The EGFR mRNA expression levels in SCC1 and 1Cc8. C) A network of EGFR pathway-associated genes determined by Ingenuity Pathways Analysis using 900 probes that are differentially expressed between cetuximab-sensitive and -resistant cell lines (SCC1 and 1Cc8). Red: genes that are expressed at higher levels in the cetuximab-resistant cell line compared to the cetuximab-sensitive cell line. (1.11 MB TIF) [file pone.0012702.s001.tif]

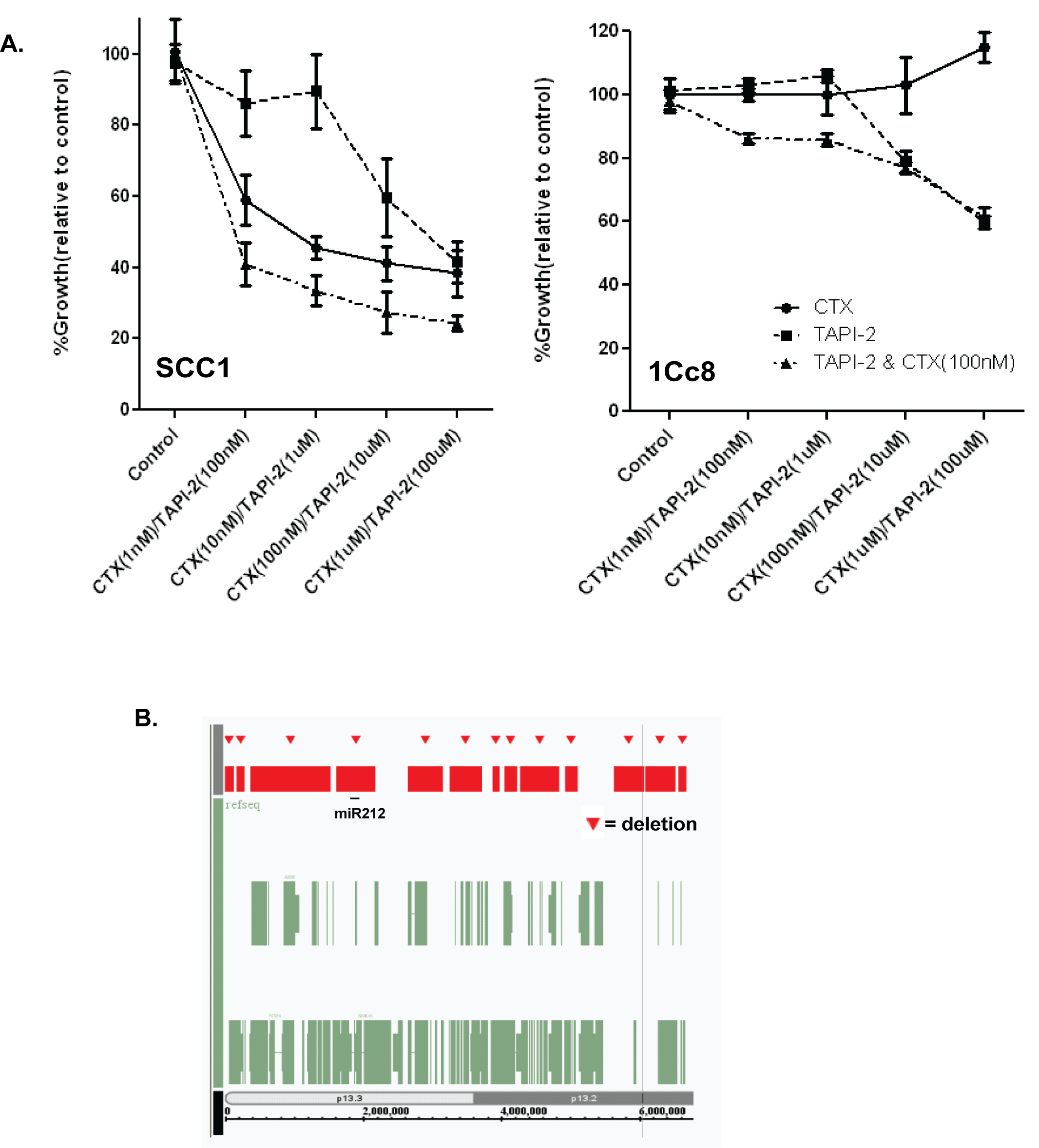

Supplement: Figure S2 — A) Growth inhibition rate of SCC1 and 1Cc8 following treatment with cetuximab, TAPI-2 or a combination of both drugs measured by MTS assay. B) Chromosomal map of 17p13.3 containing miR-212 coding region using genome wide single nucleotide polymorphism data of JHU022. (1.01 MB TIF) [file pone.0012702.s002.tif]
