# Supplementary material for: Regulation of Heparin-Binding EGF-Like Growth Factor by miR-212 and Acquired Cetuximab-Resistance in Head and Neck Squamous Cell Carcinoma
Source: PLoS One. 2010 Sep 13;5(9):e12702. doi: 10.1371/journal.pone.0012702 (PMC2938338; doi:10.1371/journal.pone.0012702)
Supplement: Table S1 — Culture media and sources of 34 head and neck cancer cell lines and HaCaT cells. (0.05 MB DOC) [file pone.0012702.s003.doc]

**Table S1.** Culture media and sources of 34 head and neck cancer cell lines and HaCaT cells.

| Cell Name | Media | Source |
| --- | --- | --- |
| SQ20B | A | University of Chicago (Ralph Weichselbaum) |
| SCC61 | A | University of Chicago (Ralph Weichselbaum) |
| SCC25 | A | American Type Culture Collection |
| FaDu | A | American Type Culture Collection |
| SQ9G | A | University of Chicago (Ralph Weichselbaum) |
| JSQ3 | A | University of Chicago (Ralph Weichselbaum) |
| SCC9 | A | American Type Culture Collection |
| SCC15 | A | American Type Culture Collection |
| OSCC3 | A | University of Chicago (Mark Lingen) |
| UMSCC047 | B | University of Michigan (Thomas Carey) |
| UPSCC090 | B | University of Pittsburgh (Robert Ferris) |
| 93VU147T | B | The Vrij University (Renske D. M. Steenbergen) |
| CAL27 | B | American Type Culture Collection |
| TU-167 | B | M.D. Anderson Cancer Center (Gary Clayman) |
| MDA1986 | B | M.D. Anderson Cancer Center (Gary Clayman) |
| JHU012 | C | The Johns Hopkins University (David Sidransky) |
| JHU013 | C | The Johns Hopkins University (David Sidransky) |
| JHU022 | C | The Johns Hopkins University (David Sidransky) |
| SKN-3 | C | Japanese Collection of Research Bioresource |
| UNC7 | D | University of North Carolina (Wendell Yarbrough) |
| UNC10 | D | University of North Carolina (Wendell Yarbrough) |
| HN5 | E | Ludwig Institute for Cancer Research |
| UMSCC6 | E | University of Michigan (Thomas Carey) |
| UMSCC1 | E | University of Michigan (Thomas Carey) |
| 1Cc8 | E | University of Wisconsin (Deric Wheeler) |
| Ho-1-N-1 | F | Japanese Collection of Research Bioresource |
| Ho-1-u-1 | F | Japanese Collection of Research Bioresource |
| T-167 | F | M.D. Anderson Cancer Center (Gary Clayman) |
| T-409 | F | M.D. Anderson Cancer Center (Gary Clayman) |
| HSC-2 | G | Japanese Collection of Research Bioresource |
| HSC-3 | G | Japanese Collection of Research Bioresource |
| Hep-2 | H | Hokkaido University (Nobuhiko Oridate) |
| DRHep-2 | H | Hokkaido University (Nobuhiko Oridate) |
| HEp2-CDDP | H | Hokkaido University (Nobuhiko Oridate) |
| HaCaT | I | Cell Lines Service Germany |

A: DMEM/F12 + 10% FBS + Hydrocortisone (0.4 μg/ml)

B: DMEM + 10% FBS

C: RPMI + 10% FBS

D: MEM + 10% FBS + 1x NEAA + 2 mM Gln + 1% Insulin-Transferrin-Selenium

E: DMEM + 10% FBS + Hydrocortisone (1 μg/ml)

F: DMEM/F12 + 10% FBS

G: MEM + 10% FBS

H: DMEM + 5% FBS

I: 80% MCDB153 + 20% L15 + 1% FBS
