# Supplementary material for: Regulation of Heparin-Binding EGF-Like Growth Factor by miR-212 and Acquired Cetuximab-Resistance in Head and Neck Squamous Cell Carcinoma
Source: PLoS One. 2010 Sep 13;5(9):e12702. doi: 10.1371/journal.pone.0012702 (PMC2938338; doi:10.1371/journal.pone.0012702)
Supplement: Table S2 — 900 probes that are differentially expressed between SCC1 and 1Cc8. (0.15 MB PDF) [file pone.0012702.s004.pdf]

Table S2. 900 probes that are differentially expressed between SCC1 and 1Cc8

Gene Title	SCC1-1	SCC1-2	SCC1-3	1CC8-1	1CC8-2	1CC8-3
keratin 13	12.27185	12.596	12.10592	7.533652	7.815657	7.550398
keratin 15	10.45992	10.95383	9.513009	6.376461	6.851977	6.310437
family with sequence similarity 101, member B	7.016914	6.771083	7.044134	3.508219	3.682148	3.165991
small proline-rich protein 1A	8.769677	8.721232	8.968737	5.078395	5.958846	5.123436
TIMP metallopeptidase inhibitor 3	9.290314	9.450275	9.160716	6.096357	5.929855	5.799584
TIMP metallopeptidase inhibitor 3	8.909781	8.947557	8.417738	5.472483	5.652503	5.341288
ankyrin repeat domain 38	8.548027	8.563866	8.868394	5.307224	5.883651	4.981968
embryonal Fyn-associated substrate	7.433701	7.616783	7.338655	4.455111	3.999668	4.345238
unc-5 homolog B (C. elegans)	8.094766	8.170169	8.062465	4.876466	4.883876	5.055383
lymphocyte antigen 6 complex, locus D	8.158842	8.489719	7.793487	5.074139	5.212085	4.74848
TIMP metallopeptidase inhibitor 3	9.23926	9.220713	9.077962	5.853861	6.085531	6.298164
lysyl oxidase	8.803211	9.512515	8.009997	5.880596	5.753882	5.481447
sciellin	7.744074	8.206273	7.594888	4.405259	5.166653	4.793039
small proline-rich protein 3	8.301928	8.440048	8.532887	5.281508	5.847798	5.331469
lysyl oxidase	7.792937	8.408061	7.356362	5.246832	4.90314	4.612488
olfactomedin-like 2A	8.663695	8.916993	8.772175	5.800106	6.105832	5.759592
AHNAK nucleoprotein 2	8.754135	9.081858	8.455972	6.252491	5.701521	5.746295
chromosome 10 open reading frame 58	9.368636	9.606519	9.023958	6.429057	6.735642	6.380216
keratin 23 (histone deacetylase inducible)	7.939844	8.538349	7.749911	4.947459	5.336424	5.516863
chromosome 10 open reading frame 58	10.17799	10.56465	10.05516	7.381839	7.690509	7.315767
kallikrein-related peptidase 10	7.856326	7.944094	7.949006	5.179011	5.282048	4.902201
keratin 4	8.650273	9.060248	8.65752	6.190123	5.923005	5.93248
CDNA clone IMAGE:4830452	6.452335	7.021905	6.347448	3.64857	3.921659	3.96827
family with sequence similarity 101, member B	7.418836	7.369041	7.463447	4.713553	4.581561	4.733608
cysteine-rich protein 2	8.469645	8.526695	8.574162	6.023582	5.661571	5.820623
synaptotagmin XVII	7.383143	7.334383	7.254645	4.528602	4.555788	4.844255
placenta-specific 2	9.01421	9.217189	8.950726	6.437618	6.396583	6.468254
fibronectin 1	11.33578	11.00719	11.2636	8.618624	8.551291	8.657634
Imprinted in Prader-Willi syndrome	7.042263	6.789682	6.906487	4.410381	4.566319	3.994819
interleukin 1 receptor antagonist	8.541103	8.824987	8.435166	5.94922	6.054292	6.039335
plakophilin 1 (ectodermal dysplasia/skin fragility syndrome)	8.317691	8.500427	8.375257	5.881481	5.747693	5.830615
Full length insert cDNA clone ZD83D05	6.586681	6.759784	6.577909	3.867547	4.62619	3.72617

plakophilin 1 (ectodermal dysplasia/skin fragility syndrome)	8.962373	8.993804	8.904339	6.307641	6.467257	6.439094
small proline-rich protein 1A	7.646943	7.356675	7.762586	4.54495	5.517062	5.102789
G protein-coupled receptor 109B	10.04232	10.36512	9.935402	7.546684	7.863797	7.37556
sciellin	6.313012	6.30687	5.812568	3.825866	4.052501	3.028207
Full length insert cDNA clone YZ38E04	7.651037	7.776718	7.511889	4.929663	5.454881	5.051854
fibronectin 1	11.52355	11.21332	11.50785	8.936851	8.92461	8.88735
chloride intracellular channel 3	9.005176	9.125928	9.05015	6.802044	6.522767	6.388924
fibronectin 1	11.49043	11.20979	11.45088	8.876637	8.933458	8.935375
inhibitor of DNA binding 2, dominant negative H-loop-H protein	7.500191	7.057542	7.374789	4.775745	4.92274	4.835136
Small nuclear ribonucleoprotein polypeptide N	7.080441	6.928292	6.730902	4.32673	4.826624	4.205133
serpin peptidase inhibitor, clade B (ovalbumin), member 9	5.632618	5.613321	5.542636	3.05035	3.170576	3.21054
mucin 16, cell surface associated	6.400712	6.406747	6.035506	3.573449	3.765145	4.191177
hypothetical protein MGC10981	8.660636	8.836982	8.865449	6.417049	6.09674	6.537805
small nuclear ribonucleoprotein polypeptide N	9.535171	9.516473	9.648636	7.042235	7.307942	7.096263
B-cell CLL/lymphoma 11A (zinc finger protein)	7.01233	7.173834	6.729542	4.323175	5.017254	4.324923
keratin 14 (epidermolysis bullosa simplex, Dowling-Meara, Koebner)	12.75941	12.73539	12.96912	10.52083	10.46209	10.24316
arsenic (+3 oxidation state) methyltransferase	5.427222	5.920726	5.460726	3.236718	3.250909	3.127707
epithelial membrane protein 1	11.11719	11.18454	10.75549	8.706692	8.609364	8.604833
fibronectin 1	11.47294	11.10285	11.39196	8.959921	8.997675	8.903415
progestin and adipoQ receptor family member V	8.079627	8.089322	7.976149	5.616478	5.825991	5.634373
BTB (POZ) domain containing 11	6.955132	7.190272	7.102952	4.679683	5.006061	4.503267
epithelial membrane protein 1	9.847277	9.949995	9.618641	7.450307	7.583206	7.329522
S100 calcium binding protein P	9.704826	9.975677	9.626475	7.476508	7.565188	7.238851
C-type lectin domain family 2, member B	7.093571	7.884471	5.6887	4.574954	4.876204	4.196825
nucleosome assembly protein 1-like 5	6.469036	6.09548	5.852531	3.561741	3.876888	3.966803
alkaline phosphatase, placental (Regan isozyme)	6.572982	6.605657	6.533551	4.692304	4.166319	3.854221
gap junction protein, beta 6	7.982902	8.234458	7.664682	5.395848	5.976506	5.532609
ephrin-B2	6.670426	6.35252	6.822858	4.519471	4.31092	4.122676
Synaptotagmin XVII	5.721794	6.266997	5.746532	3.547532	3.481964	3.834673
nebulette	7.052222	7.460395	6.995399	4.906516	5.222266	4.538252
nucleosome assembly protein 1-like 5	7.60597	7.553415	7.683671	5.124358	5.419371	5.481131
---	7.442341	7.630025	7.591918	4.631562	5.581324	5.669188
small nuclear ribonucleoprotein polypeptide N	10.3095	10.35702	10.34449	8.042404	8.105155	8.154346
---	7.030633	7.061987	7.023356	5.183061	4.762339	4.472314

acyltransferase like 1	6.202397	5.985	5.743261	4.097699	3.531211	3.606409
lysyl oxidase	6.629028	6.835561	5.985575	4.42316	4.15203	4.229719
FYN binding protein (FYB-120/130)	5.777217	6.054672	5.815498	3.575881	3.767103	3.662215
CDNA FLJ33569 fis, clone BRAMY2010317	5.729856	5.802241	6.033298	3.369643	3.464573	4.106673
imprinted in Prader-Willi syndrome	6.91866	6.985737	6.84731	4.759898	4.763684	4.639783
mitogen-activated protein kinase kinase 6	7.561004	8.015773	7.678555	5.4874	5.812181	5.374267
CDNA FLJ13457 fis, clone PLACE1003343	7.093659	7.41463	6.899028	5.077712	4.749357	4.998779
dynamin 1	7.545854	7.627943	7.687864	5.600976	5.375067	5.319495
fibroblast growth factor receptor 3	10.2926	10.45557	10.15105	8.121784	8.274527	7.94258
serpin peptidase inhibitor, clade B (ovalbumin), member 13	5.856558	6.048801	6.173825	3.67785	3.703019	4.144995
leucine rich repeat containing 34	5.765873	5.708346	5.80748	3.680958	3.504753	3.559868
Transcribed locus, moderately similar to XP_001102724.1	8.783336	8.763138	8.744662	6.768743	6.561136	6.48164
hypothetical LOC653602	5.273339	5.397593	4.678549	3.068255	3.067503	2.801765
ring finger protein 128	5.525441	6.018735	5.140989	3.423846	3.498007	3.353488
matrix metallopeptidase 12 (macrophage elastase)	6.760203	7.01664	5.854295	4.409551	4.503298	4.341434
N-myc downstream regulated gene 1	9.901411	10.7768	9.137233	7.810413	7.966208	7.680432
quinolinate phosphoribosyltransferase	9.840271	9.774437	9.716553	7.766562	7.81572	7.44956
FLJ41603 protein	7.533932	7.767723	7.441255	5.461924	5.674379	5.41769
B-cell CLL/lymphoma 11A (zinc finger protein)	6.887665	6.700677	6.606025	4.915075	4.726752	4.367174
potassium inwardly-rectifying channel, subfamily J, member 15	6.938796	6.995665	7.121647	5.301254	4.979418	4.602393
gelsolin (amyloidosis, Finnish type)	9.211539	9.390032	9.110598	7.107959	7.388816	7.069681
H19, imprinted maternally expressed untranslated mRNA	11.4464	11.50831	11.40227	9.533876	9.481054	9.206017
glutathione S-transferase A4	7.252979	7.822105	6.961656	5.578685	5.357386	4.975601
heat shock 27kDa protein 1	12.69035	12.84421	12.61632	10.77778	10.75436	10.55616
Rh family, C glycoprotein	8.60525	8.883649	8.354858	6.504316	6.960852	6.325299
serpin peptidase inhibitor, clade B (ovalbumin), member 13	5.06613	5.070393	5.147182	3.065864	3.1262	3.068994
TIMP metallopeptidase inhibitor 3	9.006386	9.188397	8.841502	6.844965	7.210254	7.013018
SMAD family member 4	7.605099	7.433768	7.537441	5.708801	5.439712	5.46645
hypothetical protein MGC16075	6.173078	6.180141	6.424001	4.274157	4.630453	3.948993
BTB (POZ) domain containing 11	8.59106	8.719212	8.769381	6.680081	6.885644	6.622822
tripartite motif-containing 29	11.43199	11.51305	11.5466	9.634127	9.616056	9.370495
transforming growth factor, beta-induced, 68kDa	11.48154	11.44517	11.57743	9.582662	9.654842	9.411131
---	7.096958	7.154877	7.088683	5.185514	5.282257	5.021242
tripartite motif-containing 29	9.120736	9.074051	9.251041	7.498422	6.985411	7.134907

UDP-N-acetyl-alpha-GalNAc-T12	7.703942	7.895767	7.797512	5.889597	6.052201	5.673209
tumor necrosis factor (ligand) superfamily, member 10	7.6203	7.926855	7.286589	5.508808	5.842834	5.709183
CDC42 effector protein (Rho GTPase binding) 5	6.744496	6.915764	6.791624	4.976249	4.707152	5.002813
layilin	7.040409	6.87416	6.998631	5.032457	5.174048	4.947792
cytochrome P450, family 2, subfamily R, polypeptide 1	6.388633	6.066638	6.121895	4.237089	4.250187	4.341315
wingless-type MMTV integration site family, member 10A	8.058379	8.028628	8.103128	6.144474	6.321443	5.989948
progestin and adipoQ receptor family member V	6.973611	6.18712	7.13313	4.713499	4.863328	4.998482
mesoderm specific transcript homolog (mouse)	9.735833	9.80593	9.784747	7.785398	8.076324	7.747527
sodium channel, nonvoltage-gated 1 alpha	9.895031	10.20234	9.902507	8.290602	8.107716	7.917177
H2A histone family, member Y2	9.389046	9.415545	9.349434	7.471933	7.513088	7.499388
sterile alpha motif domain containing 9	8.601882	8.907338	8.358575	6.565269	7.001204	6.698663
Rho-related BTB domain containing 3	8.749407	8.706729	8.66427	6.956415	6.844227	6.750163
chromosome 2 open reading frame 55	6.355548	6.43378	6.197941	4.422118	4.558415	4.446787
apolipoprotein B mRNA editing enzyme, catalytic polypeptide-like 3B	7.722388	8.026169	7.365956	5.869446	5.977313	5.729204
H19, imprinted maternally expressed untranslated mRNA	13.58842	13.62339	13.50979	11.6308	11.93501	11.62804
tumor necrosis factor (ligand) superfamily, member 10	6.3138	6.472883	5.827586	4.295831	5.006738	3.805305
MRNA; cDNA DKFZp686A11113 (from clone DKFZp686A11113)	5.194761	5.169369	4.949986	3.463573	3.03662	3.311828
Rho-related BTB domain containing 3	10.05775	10.06318	9.854053	8.222556	8.214049	8.081172
kallikrein-related peptidase 5	7.521436	7.353371	7.469357	5.897904	5.261788	5.736294
methyltransferase like 7A	9.080788	9.439468	8.924315	7.451239	7.388033	7.165226
preferentially expressed antigen in melanoma	6.928473	6.731654	6.933575	4.884897	5.190256	5.095688
GIPC PDZ domain containing family, member 2	5.594303	5.409308	5.750776	3.853612	3.685428	3.797954
Rho-related BTB domain containing 3	9.747858	9.677775	9.588616	7.950257	7.726161	7.923886
insulin-like growth factor 2 (somatomedin A)	5.976179	6.250175	5.98047	4.273607	4.122846	4.399502
Kruppel-like factor 9	5.484117	5.850849	4.739606	3.99988	3.398779	3.384611
trophoblast-derived noncoding RNA	9.476042	9.644377	9.350962	7.858836	7.718424	7.610962
similar to olfactory receptor, family 7, subfamily A, member 17	6.661465	6.421029	6.562728	4.800543	4.900395	4.676548
serum deprivation response (phosphatidylserine binding protein)	7.245682	7.416186	7.007851	5.592804	5.581835	5.235609
troponin T type 1 (skeletal, slow)	7.757896	7.872486	7.512784	6.000862	6.28955	5.606489
proprotein convertase subtilisin/kexin type 9	8.171023	7.921659	8.358033	6.554686	6.58484	6.071099
unc-5 homolog B (C. elegans)	6.373661	6.337921	6.190248	4.865382	4.436815	4.369971
acetyl-Coenzyme A acetyltransferase 2	9.590092	9.67972	9.776794	7.826618	8.073643	7.927426
tumor necrosis factor (ligand) superfamily, member 10	6.579164	6.871275	6.170536	4.960976	4.580324	4.87037
sciellin	6.237665	6.44641	6.223957	4.479911	4.81265	4.41256

retinoic acid early transcript 1E	6.989807	6.922467	7.007851	4.983998	5.494649	5.243943
zinc finger protein 711	7.053152	7.135424	6.835518	4.905854	5.533577	5.397359
thrombomodulin	8.196404	8.244023	8.037275	6.440607	6.444162	6.410586
Rho-related BTB domain containing 3	7.403661	7.239609	7.459494	5.798764	5.398383	5.740992
methyl-CpG binding domain protein 2	9.994078	10.10946	9.960166	8.250239	8.459742	8.2057
nebulette	6.730435	6.948077	6.501412	5.127084	5.065469	4.859708
leucine rich repeat containing 34	5.123721	5.370837	4.7934	3.466137	3.398779	3.333628
malic enzyme 2, NAD(+)-dependent, mitochondrial	8.454615	8.585785	8.369435	6.776357	6.876139	6.691512
protein kinase C, delta binding protein	5.746624	5.496611	5.854295	4.121096	4.315022	3.61219
cellular retinoic acid binding protein 2	8.969093	9.121408	8.872816	7.233771	7.383965	7.299581
acyltransferase like 1	5.84045	5.670174	5.747516	3.984484	4.161341	4.075546
cathepsin H	10.1985	10.26014	10.2061	8.54114	8.588537	8.500798
Early growth response 1	12.27987	12.11581	12.13971	10.5643	10.47215	10.47326
keratinocyte differentiation-associated protein	7.245062	7.019872	7.325049	5.349167	5.578023	5.643036
homer homolog 2 (Drosophila)	7.649036	7.73028	7.842753	5.996015	5.945919	6.260446
cytochrome P450, family 24, subfamily A, polypeptide 1	6.477425	6.033748	6.188228	4.634185	4.630453	4.438809
forkhead box O1	7.151435	7.159301	7.134706	5.656009	5.438649	5.364122
UDP-N-acetyl-alpha-GalNAc-T12	6.851212	6.895866	6.820768	5.32767	5.511574	4.803893
protein phosphatase 1E (PP2C domain containing)	6.883348	7.133274	6.784094	4.948081	5.612974	5.318387
trophoblast-derived noncoding RNA	9.935335	10.0813	9.637227	8.498976	8.192866	8.054457
4-aminobutyrate aminotransferase	6.927363	6.583733	6.573704	5.078561	5.287487	4.825007
mal, T-cell differentiation protein-like	9.726733	9.68033	9.767112	8.17385	8.076847	8.041708
DIP2 disco-interacting protein 2 homolog C (Drosophila)	5.719586	5.805223	5.787671	4.325919	4.28943	3.819388
FXYD domain containing ion transport regulator 3	10.94461	11.15594	10.73769	9.325167	9.469049	9.180417
quinolinate phosphoribosyltransferase	8.657878	8.870655	8.745291	7.316569	7.129181	6.976882
butyrobetaine (gamma), 2-oxoglutarate dioxygenase 1	4.991156	5.886632	4.375721	3.508073	3.440464	3.459618
RAB27B, member RAS oncogene family	7.859549	7.883905	7.634588	6.155058	6.342017	6.063113
glycoprotein (transmembrane) nmb	5.432088	5.340273	5.198422	3.235529	4.035445	3.8945
calmodulin-like 5	6.99542	7.379397	6.335503	5.345557	5.228507	5.33255
oxysterol binding protein-like 1A	8.715825	8.841681	8.811363	7.269815	7.355979	6.960605
neurofibromin 1	7.142017	7.156971	7.210874	5.368125	5.630542	5.732737
sterile alpha motif domain containing 9	7.559865	7.898212	7.447305	5.943815	6.243547	5.957901
kelch domain containing 8B	9.308552	9.626448	9.456264	8.067042	7.874979	7.708998
major histocompatibility complex, class II, DQ beta 1	5.647917	5.69177	5.358328	3.768193	4.361732	3.839146

adenylate cyclase 7	7.852787	7.846987	7.695654	6.393796	6.230611	6.042551
multiple EGF-like-domains 6	6.865096	7.119089	6.909481	5.426713	5.462646	5.288949
Trophoblast-derived noncoding RNA	5.736889	5.858496	5.555411	4.223681	4.117437	4.095975
thyroid hormone receptor, beta	6.510819	6.420501	6.641608	4.692522	5.136436	5.051704
CDNA clone IMAGE:5265056	6.346585	6.377929	6.366151	4.927954	4.690248	4.781848
integrin, beta 8	8.226231	8.237726	8.064196	6.730224	6.714515	6.393435
vang-like 2 (van gogh, Drosophila)	6.314207	6.367243	6.58812	4.660057	4.95709	4.989892
H6 family homeobox 2	6.264064	5.634452	6.03463	4.518145	4.517652	4.237734
fibronectin 1	7.008009	6.347817	7.253694	5.179011	5.137784	5.633192
serum deprivation response (phosphatidylserine binding protein)	7.405627	7.403655	7.11615	5.923505	5.747042	5.599116
inhibitor of DNA binding 2, dominant negative helix-loop-helix protein	6.07616	5.741121	5.954602	4.963063	3.475456	4.702971
serpin peptidase inhibitor, clade B (ovalbumin), member 5	10.13634	10.21723	10.07804	8.581583	8.650887	8.588905
Notch-regulated ankyrin repeat protein	10.02698	10.02051	10.09944	8.47475	8.631753	8.442595
oxysterol binding protein-like 1A	7.80557	7.884788	7.757586	6.282719	6.342617	6.238949
engulfment and cell motility 3	9.077924	9.21679	9.190869	7.758593	7.657958	7.510159
CDNA FLJ33569 fis, clone BRAMY2010317	5.039866	4.44639	4.126023	2.859733	3.143535	3.05118
chromosome 9 open reading frame 84	5.380117	5.387323	5.121035	3.464724	4.197467	3.678354
low density lipoprotein receptor-related protein 11	10.57958	10.61608	10.32575	8.990372	9.018749	9.001235
cyclin-dependent kinase inhibitor 1C (p57, Kip2)	6.437515	6.403451	5.575385	4.808197	4.422938	4.692242
acyltransferase like 1	6.392133	6.419981	6.511605	5.077038	5.103342	4.654062
early growth response 1	7.761686	7.207581	7.87799	6.585079	5.676842	6.114657
FXYD domain containing ion transport regulator 3	8.499172	8.698467	8.127756	6.709282	7.09847	7.093872
protein tyrosine phosphatase, receptor type, S	6.825948	6.781915	6.790313	5.535252	5.275898	5.205912
programmed cell death 4 (neoplastic transformation inhibitor)	9.392578	9.573251	9.021333	7.688625	8.117786	7.807802
latent transforming growth factor beta binding protein 2	9.371744	9.304029	9.497271	8.018127	7.901587	7.887156
aldehyde dehydrogenase 3 family, memberA1	7.319094	7.522551	7.166583	6.101587	5.636893	5.924129
CXXC finger 5	9.717195	9.806751	9.711103	8.35332	8.386563	8.16381
gelsolin (amyloidosis, Finnish type)	7.043816	7.148376	6.859508	5.503751	5.710091	5.51327
Full-length cDNA clone CS0DI029YM01	8.049114	8.077369	7.991483	6.603129	6.566545	6.625206
CXXC finger 5	9.584752	9.811219	9.670312	8.210255	8.44885	8.086521
stomatin	9.957426	9.952077	9.823244	8.467552	8.539427	8.405793
DNA segment on chromosome 4 (unique) 234 expressed sequence	5.79465	5.980041	6.153082	4.198333	4.789796	4.638225
PRO1073 protein	6.392926	6.501476	6.39362	5.255255	4.766316	4.968227
Chromosome 6 open reading frame 86	6.223886	6.538851	6.382669	4.797963	5.209086	4.847226

DEP domain containing 7	6.863457	6.719691	6.482164	5.221746	5.660665	4.911507
Fibronectin leucine rich transmembrane protein 2	7.517494	7.381647	7.541826	6.031871	6.09643	6.043487
DnaJ (Hsp40) homolog, subfamily C, member 15	7.314801	7.332362	7.217882	5.949219	5.9762	5.675476
protein phosphatase 1E (PP2C domain containing)	6.696409	6.897675	6.718624	5.469174	5.322045	5.277273
WAS protein family, member 3	6.850898	6.580242	6.397996	5.371666	5.255568	4.965672
erythrocyte membrane protein band 4.1-like 1	5.747356	5.88098	5.682847	4.189625	4.355865	4.533099
protein tyrosine phosphatase-like A domain containing 2	7.962765	8.193218	8.039709	6.713546	6.701631	6.575935
ataxin 1	6.383749	6.648306	5.890981	4.671715	5.291618	4.764573
stomatin	9.244143	9.366199	9.050748	7.854793	7.925858	7.689653
thyroid hormone receptor, beta	6.584821	6.669013	6.329624	5.064243	5.230549	5.100272
mucin 20, cell surface associated	7.508374	7.761591	7.1301	6.051998	6.3638	5.822799
Transcribed locus	5.703907	5.982131	5.866339	4.299449	4.546188	4.549662
zinc finger protein 207	7.248257	7.228411	6.867214	5.824967	5.817763	5.545475
pleckstrin homology domain containing, family A member 7	7.126603	7.306076	7.228805	5.740973	5.981071	5.797713
TSC22 domain family, member 3	9.270714	9.494544	8.93285	7.913253	7.954786	7.690568
membrane protein, palmitoylated 7	5.493255	5.562326	5.306341	4.317246	3.985577	3.934286
calmodulin-like 3	5.47473	5.583429	5.491622	4.038991	4.245079	4.148977
family with sequence similarity 26, member B	7.028236	6.950754	6.77455	5.637119	5.42517	5.588427
malic enzyme 2, NAD(+)-dependent, mitochondrial	6.831128	6.836964	6.59867	5.486487	5.436648	5.241521
pre-B-cell leukemia homeobox 1	5.929446	6.157165	5.741553	4.719605	4.857726	4.152408
keratin 6A /// keratin 6C	9.394777	9.472244	9.508724	8.095454	8.085309	8.096992
Transcribed locus	7.156321	7.313868	7.324215	5.959632	5.747666	5.996187
mucin 20, cell surface associated	6.214136	6.337049	5.749933	4.847442	4.800781	4.574567
programmed cell death 4 (neoplastic transformation inhibitor)	7.013793	7.305135	6.956251	5.661328	5.913652	5.623433
Rap2-binding protein 9	5.404987	5.82527	5.404499	4.013855	4.140882	4.43166
hypothetical protein LOC254848	5.414436	4.725899	5.393429	3.881499	3.706108	3.90416
solute carrier organic anion transporter family, member 3A1	6.703645	6.793595	6.542099	5.346886	5.225027	5.434855
Notch homolog 1, translocation-associated (Drosophila)	8.816105	8.901272	8.862417	7.469177	7.484543	7.597627
interferon-induced protein with tetratricopeptide repeats 1	7.713032	8.009993	7.710387	6.49303	6.704002	6.211505
Notch homolog 3 (Drosophila)	8.358198	8.492828	8.394423	7.044865	7.189807	6.985967
solute carrier organic anion transporter family, member 3A1	7.165098	7.350324	7.206918	6.003128	6.011855	5.689122
G protein-coupled receptor 115	5.58065	5.620872	5.071165	4.132507	4.0687	4.060487
hypothetical protein LOC92482	7.72899	8.016449	7.369716	6.307431	6.381101	6.423302
hypothetical protein LOC729013	8.597482	8.572858	8.485939	7.305336	7.257242	7.107516

potassium inwardly-rectifying channel, subfamily J, member 15	6.886965	7.037572	7.080956	5.717914	5.624841	5.682034
meiosis-specific nuclear structural 1	8.069174	8.068635	7.885034	6.408119	7.01391	6.622823
family with sequence similarity 70, member A	7.029408	7.07674	6.700562	5.820475	5.382935	5.627777
hypothetical protein LOC203107	7.859628	7.782185	7.599315	6.499354	6.544015	6.230141
HECT domain containing 2	5.444945	5.516413	5.536486	4.348744	4.259834	3.924174
metallothionein 1M	5.787876	5.403243	5.812959	4.306332	4.294552	4.443264
chromodomain helicase DNA binding protein 2	6.667923	6.798876	6.501545	5.586079	5.376407	5.067739
CXXC finger 5	8.912501	9.004295	8.996353	7.663966	7.720685	7.597682
forkhead box F2	5.815145	5.65677	6.086016	4.677761	4.695843	4.262959
B-cell CLL/lymphoma 11A (zinc finger protein)	7.248436	7.319369	7.25168	5.920011	6.152873	5.839417
inhibitor of DNA binding 1, dominant negative helix-loop-helix protein	13.34943	13.23428	13.31298	12.08843	12.01107	11.89618
ATPase, Ca++ transporting, plasma membrane 4	6.750502	6.931357	6.61593	5.518372	5.815803	5.07256
BTB (POZ) domain containing 3	7.248991	7.271496	7.260185	6.07196	5.785778	6.032985
metastasis suppressor 1	9.901472	9.929837	9.79076	8.540897	8.728128	8.474855
malic enzyme 2, NAD(+)-dependent, mitochondrial	8.152942	8.168045	8.097036	6.734142	7.105036	6.703478
chromosome 12 open reading frame 59	7.837566	7.888012	7.719511	6.584327	6.591252	6.40436
tripartite motif-containing 22	6.274093	6.650311	5.88721	4.950868	5.491549	4.508764
hypothetical protein LOC652968	7.575338	7.847748	7.456701	6.597194	6.165209	6.257762
zinc finger protein 207	8.358708	8.324291	8.240736	7.085731	6.831388	7.151475
metastasis associated lung adenocarcinoma transcript 1	7.336321	7.34241	7.267147	6.155057	5.911419	6.034632
CD109 molecule	9.086945	9.160116	8.962275	7.677216	7.947525	7.743833
interleukin 20 receptor beta	7.789368	8.069251	7.750603	6.642084	6.845888	6.289073
tripartite motif-containing 7	10.49539	10.4844	10.6174	9.120403	9.434232	9.218061
hairless homolog (mouse)	9.159992	8.818243	9.021069	7.889147	7.574627	7.713751
Transcribed locus	4.917036	5.080969	4.833842	3.773572	3.780379	3.456233
leucine zipper transcription factor-like 1	7.515216	7.961447	7.605268	6.398355	6.48827	6.380113
tumor suppressor candidate 1	7.536743	7.537886	7.695275	6.357068	6.345691	6.260473
inhibitor of DNA binding 4, dominant negative helix-loop-helix protein	4.866811	4.339618	4.769568	3.482106	3.460596	3.236601
chromosome 6 open reading frame 85	8.185559	8.233322	7.981273	6.908852	6.870905	6.837936
cystatin A (stefin A)	11.63503	11.73757	11.5191	10.41367	10.45147	10.25319
keratin 6A /// keratin 6B /// keratin 6C	8.952015	8.89255	9.007059	7.614104	7.783392	7.685995
programmed cell death 4 (neoplastic transformation inhibitor)	6.895967	6.960299	6.648043	5.507751	5.865802	5.366013
MAX interactor 1	9.223564	9.720974	8.859678	8.11647	8.045759	7.878119
ovary-specific acidic protein	6.527293	6.311052	6.026379	5.158377	4.785675	5.176521

phospholipase D1, phosphatidylcholine-specific	5.874405	6.359272	6.158279	4.785781	4.780813	5.093056
serpin peptidase inhibitor, clade B (ovalbumin), member 7	7.8169	7.723287	7.563326	6.530577	6.511795	6.350244
potassium channel tetramerisation domain containing 15	6.350144	6.213127	6.376023	5.164989	5.239871	4.829246
Homo sapiens, clone IMAGE:4401608, mRNA	5.939407	5.901251	5.854979	4.644419	5.059397	4.286797
dickkopf homolog 3 (Xenopus laevis)	8.079581	8.113388	8.04655	7.039248	6.784081	6.714396
synaptogyrin 1	7.781816	8.005388	7.907582	6.784506	6.606383	6.606567
CDNA FLJ39926 fis, clone SPLEN2021157	8.224552	8.154979	8.649519	7.239012	7.164855	6.932915
thrombomodulin	7.372214	7.40649	7.113141	6.0367	6.077413	6.087774
chromosome 1 open reading frame 116	6.459607	6.497674	6.649419	4.954712	5.420625	5.546843
ATP-binding cassette, sub-family A (ABC1), member 12	5.465788	5.655475	5.091423	4.233873	4.026431	4.272645
family with sequence similarity 62 (C2 domain containing) member B	7.358067	7.316177	7.166345	6.218008	5.97551	5.967626
Homo sapiens, clone IMAGE:3883659, mRNA	5.438938	5.401202	5.613095	4.423634	4.341572	4.021801
CDNA FLJ31650 fis, clone NT2RI2004079	5.0873	4.902169	4.649052	3.553917	3.875002	3.551041
protein tyrosine phosphatase, non-receptor type 13	7.759202	7.596737	7.395696	6.340696	6.401284	6.365522
CDNA clone IMAGE:5261375	4.66615	4.447941	4.267058	3.376444	3.151561	3.213475
dishevelled associated activator of morphogenesis 1	9.257662	9.06549	8.955439	7.870552	7.91258	7.856813
ankyrin repeat domain 41	4.893948	4.804142	5.174712	3.855867	3.80338	3.58112
oxidized low density lipoprotein (lectin-like) receptor 1	9.059511	8.889277	9.046705	7.797079	7.942052	7.62648
A kinase (PRKA) anchor protein (yotiao) 9	5.185099	5.486509	5.026428	3.952566	4.191748	3.931186
---	5.674873	5.947549	5.587691	4.628679	4.594186	4.367747
Full length insert cDNA clone YZ04E02	4.513457	4.801079	4.564369	3.459169	3.642063	3.16091
creatine kinase, brain	8.114549	8.338042	8.283892	7.361189	6.799558	6.964394
ERBB receptor feedback inhibitor 1	9.842611	9.99017	9.710361	8.627984	8.722599	8.581806
adenylosuccinate synthase like 1	7.108137	7.479195	6.920979	6.063828	5.837403	5.996603
phospholipase D1, phosphatidylcholine-specific	6.546512	6.846874	6.049285	5.460855	5.357766	5.017417
collagen, type XXVII, alpha 1	6.59547	6.601005	6.575291	5.393604	5.636205	5.142492
similar to Peroxisomal coenzyme A diphosphatase NUDT7	7.280361	7.50416	7.203413	6.356604	6.226079	5.807956
family with sequence similarity 26, member B	6.80341	6.748106	6.329204	5.43416	5.487334	5.364239
prostaglandin E synthase	9.901606	9.802464	10.07697	8.755564	8.83579	8.609484
cytochrome b reductase 1	8.468842	8.540731	8.100764	7.291667	7.134283	7.105978
G protein-coupled receptor 115	7.00715	7.201625	7.065721	5.888962	6.089002	5.722225
inhibitor of DNA binding 3, dominant negative helix-loop-helix protein	11.06729	10.84062	11.10688	9.981709	9.751005	9.710524
forkhead box O1	6.82037	6.868958	6.83929	5.859071	5.747643	5.353179
feline leukemia virus subgroup C cellular receptor family, member 2	6.076852	5.99045	6.102448	4.931007	4.853594	4.818041

crystallin, alpha B	6.796359	7.030986	6.442089	5.5323	5.771429	5.401734
eukaryotic translation initiation factor 2C, 4	6.397597	6.490619	6.285257	5.210643	5.56739	4.833315
potassium channel tetramerisation domain containing 15	7.077943	6.980506	6.945816	5.80431	5.898464	5.740514
eukaryotic translation initiation factor 4E family member 3	6.765162	6.936194	6.598572	5.41332	5.672626	5.658799
phosphoribosyl transferase domain containing 1	6.249887	6.50007	6.154526	5.193177	5.364659	4.793039
Chromodomain helicase DNA binding protein 2	6.024706	6.421409	5.904178	5.091252	4.955415	4.756083
sterile alpha motif domain containing 9-like	6.403964	7.044286	6.684976	5.069851	5.825841	5.695364
collagen triple helix repeat containing 1	6.492492	6.598004	6.400372	5.454291	5.343284	5.16389
FAT tumor suppressor homolog 2 (Drosophila)	9.298532	9.098061	9.264287	8.151056	8.115643	7.867157
ryanodine receptor 1 (skeletal)	5.760128	5.62114	6.097946	4.692522	5.038688	4.232889
complement component 1, s subcomponent	6.913519	7.137865	6.730375	5.715048	5.815863	5.736523
endothelial differentiation, sphingolipid G-protein-coupled receptor, 8	7.187872	7.032063	7.338427	6.056588	5.91403	6.075375
Transcribed locus	4.459229	4.953125	4.77775	3.564058	3.344967	3.770035
CD44 molecule (Indian blood group)	7.00715	7.18064	6.87193	5.885063	5.753882	5.918983
neurofibromin 1	6.579764	6.92433	6.670177	5.383124	5.601442	5.688454
SET domain containing 6	7.283678	7.218704	7.166152	6.147279	5.965765	6.054367
parvin, beta	6.137287	6.418637	6.52274	5.073752	5.327878	5.17779
latent transforming growth factor beta binding protein 2	7.66706	7.336268	7.415508	6.294776	6.359053	6.272934
BCL2/adenovirus E1B 19kDa interacting protein 3-like	8.699981	9.002847	8.364573	7.435142	7.700115	7.44208
premature ovarian failure, 1B	4.833358	4.958925	4.213507	3.505516	3.814411	3.199729
nudix (nucleoside diphosphate linked moiety X)-type motif 13	7.606288	7.541021	7.389323	6.550652	6.43996	6.060809
2',5'-oligoadenylate synthetase 1, 40/46kDa	6.521613	6.726652	6.22907	5.402822	5.206713	5.388305
chromosome 11 open reading frame 71	6.453354	6.911068	6.676214	5.533935	5.566629	5.464622
elongation factor Tu GTP binding domain containing 1	8.376805	8.476415	8.328268	6.938888	7.473118	7.300467
AT rich interactive domain 5B (MRF1-like)	8.003637	8.242356	7.839991	6.835659	6.994815	6.790389
complement component 1, r subcomponent	8.263211	8.368961	8.213435	7.290461	7.186828	6.904856
chromosome 1 open reading frame 116	7.260319	7.356401	7.136897	6.07054	6.290496	5.931301
cadherin, EGF LAG seven-pass G-type receptor 2	8.314603	8.258936	8.369401	7.162933	7.277336	7.046169
selenium binding protein 1	5.20658	5.24126	4.748309	3.887859	3.874935	3.979683
hypothetical protein LOC283278	6.549379	6.448503	6.429965	5.273013	5.473125	5.229215
Kruppel-like factor 5 (intestinal)	9.038638	9.310849	8.969221	7.988907	7.858823	8.018573
Rap guanine nucleotide exchange factor (GEF)-like 1	7.814453	7.965006	7.749384	6.59421	6.741488	6.746433
hypothetical protein LOC147645	5.467137	5.469378	5.680081	4.262731	4.292207	4.626038
glycosyltransferase 8 domain containing 2	8.652044	8.77462	8.80736	7.671247	7.421404	7.708254

HMG-box transcription factor 1	8.864994	9.038284	8.609421	7.656567	7.782486	7.640937
prominin 2	8.926464	8.934091	8.624696	7.834013	7.709391	7.515836
phospholipase D1, phosphatidylcholine-specific	5.834307	6.240377	5.7993	4.574777	5.154199	4.723319
hCG1818231	5.8428	6.168875	5.678015	4.855402	4.507571	4.912784
sal-like 2 (Drosophila)	7.33362	6.868958	7.132216	5.849407	5.989353	6.085426
fibroblast growth factor receptor 3	5.836176	5.93797	5.879179	4.603589	4.58667	5.052852
GABA(A) receptor-associated protein like 1	9.076645	9.288698	9.006436	8.069732	8.023443	7.874581
hypothetical protein LOC283404	7.223809	7.581283	7.231661	6.239029	6.331841	6.064495
endothelial differentiation, sphingolipid G-protein-coupled receptor, 8	9.49823	9.489413	9.7072	8.51806	8.427257	8.356594
SVOP-like	4.448988	4.932822	4.732451	3.341743	3.885691	3.498386
transmembrane 4 L six family member 1	4.300341	4.856478	4.152415	2.871677	3.864022	3.19347
4-aminobutyrate aminotransferase	6.475642	6.373743	6.783549	5.395569	5.476211	5.38124
BCL2-like 10 (apoptosis facilitator)	5.022907	4.751823	4.658334	3.806594	3.69595	3.556086
chromosome 1 open reading frame 116	5.698224	5.993265	5.825094	4.750299	4.794444	4.598633
hypothetical protein FLJ31033	7.372598	7.195889	7.267391	6.180445	6.269193	6.024044
proline dehydrogenase (oxidase) 1	7.398303	7.635482	7.314872	6.699803	6.222345	6.065463
CDNA FLJ25706 fis, clone TST04817	8.939093	8.95921	8.965195	8.074033	7.483954	7.959816
Transcribed locus, moderately similar to XP_517655.1	6.189952	6.806407	6.346169	5.728235	5.035719	5.238996
ephrin-B3	6.203968	6.367243	6.175755	5.15628	5.008637	5.24883
TBC1 domain family, member 4	7.756787	7.957823	7.698284	6.730287	6.683444	6.671401
nicotinamide nucleotide adenylyltransferase 2	5.909899	5.210033	5.637836	4.633071	4.718179	4.08062
eukaryotic translation initiation factor 4E family member 3	5.655581	5.683654	5.263704	4.653326	4.197101	4.431661
cysteine-rich protein 1 (intestinal)	5.611881	4.989315	5.055463	4.178518	4.079107	4.079587
DNA-damage-inducible transcript 4	11.59559	11.86581	11.34124	10.68025	10.45709	10.37126
BCL2/adenovirus E1B 19kDa interacting protein 3-like	8.794418	9.005137	8.517372	7.653783	7.826697	7.542411
transmembrane protein 68	9.333349	9.359559	9.345172	8.329113	8.251111	8.167686
prostaglandin E synthase	8.557591	8.416931	8.691648	7.423707	7.445154	7.510008
premature ovarian failure, 1B	5.6153	5.459535	5.220796	4.606246	4.394604	4.010884
carnitine O-octanoyltransferase	5.867414	5.517401	5.567362	4.66757	4.700916	4.302737
chromosome 11 open reading frame 52	7.840549	8.061074	7.79948	6.837694	6.80228	6.783418
transmembrane protease, serine 11E	5.016127	5.080445	5.112023	4.249014	3.684953	4.005123
cytochrome c, somatic	7.697672	7.506228	7.78178	6.530922	6.724624	6.461085
CDNA FLJ31475 fis, clone NT2NE2001598	5.648177	5.519872	5.457688	4.71817	4.079347	4.560317
phosphodiesterase 9A	5.729262	5.619907	5.97045	4.894461	4.413553	4.748254

ribonuclease, RNase A family, 4	5.119719	6.12208	5.026346	4.088643	4.546999	4.369272
FLJ25476 protein	9.335886	9.372534	9.4033	8.391558	8.314792	8.142331
glypican 1	8.924536	8.853213	9.09976	7.907619	7.925358	7.789255
early growth response 1	10.3777	10.32966	10.30667	9.199125	9.095715	9.464051
spastic paraplegia 11 (autosomal recessive)	8.991178	9.070084	8.828918	7.778582	8.003806	7.857285
transmembrane 4 L six family member 1	4.686278	4.675894	4.574838	3.97298	3.666947	3.047272
spindlin family, member 3	5.523655	5.493088	5.526861	4.417552	4.926846	3.949459
Glucocorticoid receptor alpha mRNA, variant 3' UTR	5.44142	5.943793	5.38852	4.640397	4.547396	4.337742
neurofibromin 1	6.070588	6.043111	6.03419	4.949868	4.651727	5.298677
UDP-N-acetyl-alpha	7.207379	7.313699	7.315865	6.05421	6.272063	6.26431
Transcribed locus	6.590447	6.681825	6.405683	5.408345	5.465893	5.558593
GNAS complex locus	4.973366	5.235074	5.033837	3.643766	4.233034	4.121274
growth arrest-specific 6	6.268671	6.570116	6.526341	5.434159	5.059519	5.627433
tripartite motif-containing 7	6.372002	6.416149	6.507165	5.800228	5.3232	4.928781
chromosome 1 open reading frame 21	8.726089	8.824662	8.659291	7.70269	7.751137	7.516606
ral guanine nucleotide dissociation stimulator	8.449269	8.678646	8.191045	7.586626	7.354259	7.141535
matrilin 2	6.651534	6.583611	6.59016	5.719828	5.783143	5.091501
CDNA FLJ13266 fis, clone OVARC1000960	5.684423	5.837174	5.558892	4.860042	4.402399	4.591035
interferon regulatory factor 5	8.351798	8.443042	8.606132	7.604937	7.426007	7.143069
Dipeptidyl-peptidase 7	8.999399	8.966196	9.072823	7.93919	7.963162	7.91177
major histocompatibility complex, class II, DQ beta 1	6.043314	6.388566	6.00547	4.793519	5.470641	4.951194
transducin-like enhancer of split 1	8.098007	8.091382	8.038134	6.968746	7.193933	6.84348
solute carrier family 47, member 2	6.233432	6.551757	6.223772	5.276252	5.31662	5.196472
arrestin domain containing 4	6.737994	6.669315	6.285223	5.330587	5.630351	5.513619
CXXC finger 6	5.792506	6.069549	5.934908	4.731591	5.038679	4.813177
KIAA1729 protein	6.221441	6.407066	6.311194	4.924565	5.614581	5.187552
myosin VA (heavy chain 12, myoxin)	7.714108	7.635715	7.67807	6.453794	6.723284	6.639499
keratin 5	12.43358	12.44175	12.503	11.46047	11.47453	11.23616
trophoblast-derived noncoding RNA	5.951636	6.266997	5.905788	5.106296	4.731147	5.08244
DNA (cytosine-5-)-methyltransferase 3 beta	8.350251	8.376985	8.189981	7.278815	7.261843	7.182013
metastasis associated lung adenocarcinoma transcript 1	5.88823	6.177732	6.382425	4.975581	5.125263	5.158741
major histocompatibility complex, class II, DQ beta 1	6.759097	7.12192	7.058453	5.734982	5.959203	6.05907
interleukin 1 receptor antagonist	6.183132	6.379601	6.223179	4.928004	5.606133	5.076105
D site of albumin promoter (albumin D-box) binding protein	6.753341	6.999421	6.569643	5.544098	5.902073	5.70087

CD109 molecule	8.912484	8.908501	8.728617	7.971334	7.717713	7.685994
coiled-coil domain containing 74A	5.836357	5.088634	5.95854	4.343273	4.698595	4.67091
DIP2 disco-interacting protein 2 homolog C (Drosophila)	7.040976	7.208139	7.116268	5.765967	6.14365	6.289073
leucine rich repeat containing 62	8.173838	7.846911	8.195663	6.949702	7.099697	7.004115
transglutaminase 1	7.21564	7.435268	7.293791	6.602601	6.174985	6.004734
hCG20426	7.380439	7.628222	7.451222	6.446045	6.233629	6.618088
collagen, type I, alpha 1	8.06146	8.02675	7.907598	7.207815	6.844314	6.791041
neurofibromin 1	6.696264	6.795688	6.624863	5.548069	5.76946	5.647429
spermatogenesis associated 6	4.729969	4.953737	5.302493	4.003945	3.812026	4.0218
hypothetical protein FLJ31818	6.949591	7.176536	6.945816	5.969063	6.26367	5.691079
Transcribed locus, strongly similar to XP_001095617.1	5.49398	5.230806	5.42403	4.406733	4.698596	3.900066
TBC1 domain family, member 4	7.565575	7.433466	7.33943	6.345071	6.446296	6.408342
PHD finger protein 20-like 1	6.659251	6.57386	6.5673	5.575953	5.667223	5.420641
piggyBac transposable element derived 4	5.77506	5.908529	6.138339	5.01256	5.048978	4.630211
v-fos FBJ murine osteosarcoma viral oncogene homolog	6.613337	6.617628	6.59369	5.592211	5.568371	5.535682
TBC1 domain family, member 8 (with GRAM domain)	8.227992	8.46701	7.979914	7.153135	7.34975	7.045558
kringle containing transmembrane protein 1	7.667588	7.62785	7.624415	6.596206	6.511046	6.687529
ATP binding domain 4	8.528253	8.688068	8.670881	7.528312	7.673966	7.561322
secretory leukocyte peptidase inhibitor	8.702574	8.833752	8.637443	7.721933	7.862593	7.466354
CDNA clone IMAGE:4812643	5.093041	5.502096	4.684746	4.226205	3.941226	4.000261
Clone IMAGE:119716, mRNA sequence	6.879821	6.831028	6.812754	6.008212	5.674014	5.737713
stearoyl-CoA desaturase (delta-9-desaturase)	10.07712	9.920555	10.18727	9.136592	8.997423	8.951589
spastic paraplegia 3A (autosomal dominant)	7.096944	7.148879	6.926528	6.139483	6.154366	5.779645
cadherin, EGF LAG seven-pass G-type receptor 2	7.945645	7.875561	7.892901	7.118839	6.811038	6.68753
hect domain and RLD 2 pseudogene 3	8.189222	8.133921	8.111187	7.187337	7.165868	6.986103
EPM2A (laforin) interacting protein 1	8.39356	8.563988	8.443047	7.391801	7.572153	7.347394
AT rich interactive domain 4A (RBP1-like)	6.23262	6.150412	5.984988	5.067747	5.181313	5.030218
protein-L-isoaspartate (D-aspartate) O-methyltransferase domain 1	8.462293	8.697368	7.972935	7.410207	7.529363	7.110276
frequenin homolog (Drosophila)	7.388809	7.277629	7.678176	6.394262	6.451535	6.41632
Transcribed locus	5.330897	5.508765	5.210416	4.07549	4.637195	4.263059
thrombomodulin	5.511139	5.417672	5.092982	3.99145	4.662936	4.297108
DiGeorge syndrome critical region gene 14	7.434371	7.468376	7.676487	6.647589	6.393277	6.468951
small proline-rich protein 1A	8.004251	7.93314	7.974371	6.841771	7.131732	6.872952
neuropeptide W	4.74163	4.477191	4.76765	3.791249	3.532202	3.599972

---	5.969569	6.09404	5.777017	5.256836	4.649282	4.872694
phosphorylase kinase, beta	8.841829	8.995127	8.8574	7.77633	8.100099	7.758631
metastasis associated lung adenocarcinoma transcript 1	6.128363	6.295547	5.923325	5.105015	4.96005	5.225315
CDNA FLJ41489 fis, clone BRTHA2004582	6.233837	6.756146	6.064845	5.508549	5.566645	4.923018
ankyrin 3, node of Ranvier (ankyrin G)	7.453606	7.856519	7.251949	6.622782	6.566913	6.316153
KIAA1345 protein	7.46023	7.470239	7.301887	6.268121	6.582438	6.330619
deltex 4 homolog (Drosophila)	8.362925	8.498303	8.29162	7.539473	7.407162	7.158474
Transcribed locus, moderately similar to XP_517655.1	8.647181	8.678506	8.562009	7.636993	7.603185	7.60087
gap junction protein, beta 2, 26kDa	7.488268	7.79718	6.965864	6.376317	6.552444	6.279583
CTD (carboxy-terminal domain, RNA polymerase II, polypeptide A)	7.451677	7.387306	7.294912	6.179576	6.611729	6.313755
tripartite motif-containing 34	7.354705	7.613176	7.396968	6.418129	6.465938	6.456138
Transcribed locus	5.399489	5.592222	5.617293	4.317355	4.566994	4.700978
carboxyl ester lipase (bile salt-stimulated lipase)	7.449144	8.06916	6.760034	6.681688	6.276155	6.297168
LAG1 homolog, ceramide synthase 4	5.818507	6.130247	6.031404	4.708083	5.440078	4.809937
MRNA; cDNA DKFZp564M243 (from clone DKFZp564M243)	8.09798	8.121224	8.049598	6.962236	7.222344	7.06239
Coiled-coil domain containing 91	5.467203	5.699974	5.751935	4.674867	4.582457	4.640435
Myeloid/lymphoid or mixed-lineage leukemia	6.374089	6.423511	6.221006	5.336388	5.432856	5.233372
churchill domain containing 1	7.705626	7.766605	7.675635	6.510897	6.805883	6.817496
CDNA FLJ11745 fis, clone HEMBA1005526	7.697762	7.908083	7.780597	6.810032	6.789968	6.774283
---	5.976179	5.883902	5.601452	5.063368	4.663358	4.7236
transmembrane protein 159	6.834778	6.838597	6.795336	5.873435	5.773599	5.810829
KRAB-A domain containing 2	6.407856	6.588491	6.414804	5.352144	5.361004	5.692067
ring finger protein 157	5.49605	5.499255	5.640477	4.583008	4.428423	4.620873
---	4.693372	4.552889	4.307066	5.878097	5.346208	5.33136
follistatin	6.367435	6.148655	6.300046	7.161222	7.279906	7.380324
sprouty homolog 2 (Drosophila)	6.020817	5.955403	5.809203	7.086857	6.910069	6.794867
SLIT-ROBO Rho GTPase activating protein 2 pseudogene 1	5.375554	5.144856	5.08391	6.211354	6.245631	6.155993
CD55 molecule, decay accelerating factor for complement	7.235843	7.273704	6.949315	8.198393	8.203102	8.068067
collagen, type IV, alpha 1	6.540915	6.566221	6.694466	7.650383	7.745268	7.418448
carboxymethylenebutenolidase homolog (Pseudomonas)	8.430504	8.556795	8.553153	9.61496	9.523404	9.418139
CDNA FLJ38419 fis, clone FEBRA2009846	4.571077	4.736264	4.912824	5.90961	5.529426	5.800684
RAB3B, member RAS oncogene family	8.213717	8.311355	8.43476	9.242162	9.288411	9.449965
monoglyceride lipase	8.121526	8.061127	8.188467	9.190858	9.070533	9.130828
chromosome 11 open reading frame 17	9.063145	8.901368	9.22231	10.10829	9.989151	10.12199

myosin, light chain 9, regulatory	8.602707	8.410475	8.808713	9.792259	9.530808	9.535477
protein tyrosine phosphatase, receptor-type, Z polypeptide 1	3.292104	3.19796	3.440514	4.632535	4.234031	4.103753
---	9.119816	8.792012	8.947731	10.20084	9.549683	10.15928
v-ets erythroblastosis virus E26 oncogene homolog 1 (avian)	8.553929	8.269526	8.470256	9.442813	9.372223	9.5296
CDNA FLJ33772 fis, clone BRSSN2000175	5.438744	5.784163	5.874559	6.493896	6.748484	6.906651
cytochrome P450, family 26, subfamily A, polypeptide 1	4.0771	4.344454	4.463614	5.480258	5.497432	4.959115
TRIO and F-actin binding protein	5.893281	6.20184	5.688396	7.012013	6.959853	6.866062
keratin 8	10.37964	10.19219	10.55806	11.50742	11.34993	11.32714
interleukin 15	6.155346	5.990898	5.683304	7.082917	6.990884	6.811282
transmembrane protein 118	6.603685	6.771896	6.865734	7.853275	7.751235	7.693955
solute carrier family 39 (zinc transporter), member 14	8.59959	8.280248	8.617465	9.591067	9.470321	9.495103
pleckstrin homology-like domain, family A, member 1	6.814407	6.662877	6.972815	7.893443	7.778925	7.837368
excision repair cross-complementing rodent repair deficiency	4.882765	4.801436	5.034039	5.908557	5.882563	5.98747
carbohydrate (N-acetylglucosamine-6-O) sulfotransferase 2	4.693764	4.838708	4.855642	5.935543	5.626441	5.886809
transmembrane protein 125	7.715511	7.712745	7.942888	8.851824	8.728826	8.86097
arginase, type II	8.454346	8.372132	8.492176	9.464408	9.505306	9.421211
zinc finger protein 346	3.768286	3.83385	3.413647	4.766729	4.891455	4.437321
pleckstrin homology domain containing, family A member 6	5.953552	5.398324	5.912554	6.7917	7.047134	6.509381
dpy-19-like 3 (C. elegans)	6.604114	6.291012	6.3311	7.371686	7.48989	7.449252
RGM domain family, member B	7.89501	7.836753	7.865627	8.866186	8.924349	8.893542
mediator complex subunit 24	8.275938	8.053277	8.426107	9.320902	9.189337	9.333071
Transcribed locus	5.803611	6.015882	5.919007	6.98099	7.049882	6.796029
adaptor-related protein complex 1, sigma 3 subunit	7.319262	7.26515	7.361857	8.266726	8.452601	8.318806
amidohydrolase domain containing 1	4.841853	5.054281	4.959397	5.996162	5.929254	6.022776
neuregulin 1	4.108016	3.793051	4.594825	5.22228	5.054776	5.315989
coiled-coil domain containing 92	7.553195	7.425308	7.747046	8.642575	8.713218	8.473331
solute carrier organic anion transporter family, member 4A1	7.117012	6.691947	7.26746	8.116556	8.027757	8.036925
Transcribed locus	7.085394	7.043415	7.014662	8.233473	8.05568	7.962031
solute carrier family 2 (facilitated glucose transporter), member 13	3.54511	3.529237	3.567081	4.578023	4.698595	4.482946
tumor necrosis factor receptor superfamily, member 21	6.298181	6.237697	6.274945	7.362914	7.336488	7.230457
CDNA clone IMAGE:5277883	4.389709	4.357435	3.976027	4.83026	5.510221	5.501908
neuropilin 1	4.981856	4.768881	4.811317	5.556076	6.000041	6.128766
solute carrier family 41, member 1	8.135976	8.108909	8.231712	9.277189	9.169815	9.160826
integrin, beta 4	8.492982	8.206788	8.71494	9.662993	9.283596	9.601027

polymerase (RNA) III (DNA directed) polypeptide C (62kD)	8.292414	8.205582	8.267531	9.361876	9.311886	9.224716
hypothetical protein LOC286044	4.849002	4.399694	4.266203	5.269905	5.725226	5.653406
---	5.441766	5.251803	5.238503	6.348495	6.644264	6.073053
zinc finger protein 124	5.283591	5.113842	5.088387	6.028607	6.298998	6.294193
integrin, alpha 3 (antigen CD49C, alpha 3 subunit of VLA-3 receptor)	8.713988	8.384562	8.775687	9.696531	9.696367	9.618585
solute carrier family 16, member 6 (monocarboxylic acid transporter 7)	3.746076	4.024304	3.771612	4.99644	4.954079	4.729832
tetraspanin 14	7.563444	7.597221	7.582652	8.480331	8.701667	8.706118
FYN oncogene related to SRC, FGR, YES	8.439704	8.415622	8.39987	9.571752	9.266014	9.566367
adaptor-related protein complex 1, sigma 3 subunit	6.596222	6.443892	6.546875	7.731824	7.411491	7.597144
transmembrane protein 5	8.700737	8.635289	8.664784	9.627598	9.8169	9.716076
Homo sapiens, clone IMAGE:3604678, mRNA	4.950071	4.642662	5.148486	5.968526	6.003924	5.929476
synaptotagmin-like 3	6.861472	7.009384	6.985687	8.083437	8.038157	7.896231
chemokine (C-X-C motif) ligand 3	4.85216	4.72221	4.875605	6.105145	5.753881	5.754231
solute carrier family 7 , member 2	6.539449	6.353824	6.530684	7.327277	7.710738	7.553102
transmembrane and tetratricopeptide repeat containing 2	5.170739	5.4198	5.521285	6.301814	6.593919	6.388749
thioesterase superfamily member 4	5.819159	5.934716	5.72164	6.931253	6.804886	6.915113
polymerase (DNA directed), mu	5.695257	5.719562	5.743261	7.042235	6.592404	6.700404
transforming, acidic coiled-coil containing protein 1	5.77485	5.589284	5.892307	6.697523	6.823234	6.913846
phospholipase A2-activating protein	7.902444	7.877252	8.103943	9.064602	8.978622	9.018822
chromosome 9 open reading frame 30	9.16617	9.143866	9.153807	10.3019	10.178	10.16295
cornichon homolog 3 (Drosophila)	3.297272	3.141742	3.373359	4.266904	4.170146	4.555788
MRNA; cDNA DKFZp564H1663 (from clone DKFZp564H1663)	4.338941	4.435526	3.95492	5.225952	5.536713	5.15677
vitamin D (1,25- dihydroxyvitamin D3) receptor	7.449118	7.648409	7.793086	8.720011	8.714128	8.659995
CUG triplet repeat, RNA binding protein 2	3.846698	3.328597	3.949246	4.315324	4.926524	5.086534
transforming, acidic coiled-coil containing protein 1	7.415306	7.377324	7.508374	8.382208	8.586679	8.536706
EPH receptor A2	8.091116	7.709115	8.240033	9.012154	9.061434	9.178123
schlafen family member 5	4.969632	4.668777	4.882058	5.984672	5.719563	6.029657
osteopetrosis associated transmembrane protein 1	6.451455	6.60584	6.429477	7.588113	7.679306	7.434365
coagulation factor II (thrombin) receptor-like 1	8.821645	8.78367	8.780442	9.951981	9.805312	9.845882
ELK4, ETS-domain protein (SRF accessory protein 1)	4.215053	4.443402	4.26967	5.571235	5.273012	5.30751
TRIO and F-actin binding protein	9.196777	9.272996	9.348511	10.36822	10.4011	10.2859
methionine sulfoxide reductase B3	6.434812	6.406747	6.563078	7.653998	7.482729	7.508377
arylsulfatase G	4.109709	3.89162	3.780321	5.486476	4.819383	4.728671
adrenergic, beta-2-, receptor, surface	9.606357	9.304523	9.628971	10.69312	10.49091	10.60965

tuftelin 1	8.777184	8.698871	8.978535	9.941141	9.824279	9.943009
ERO1-like beta (S. cerevisiae)	3.451838	4.173731	3.337377	4.72182	4.877386	4.618725
FYN oncogene related to SRC, FGR, YES	4.811585	4.904812	4.553996	5.701261	5.980052	5.849774
colony stimulating factor 2 receptor, alpha, low-affinity	4.402351	4.104375	3.974417	5.269906	5.279737	5.192604
thioesterase superfamily member 4	5.794315	5.852383	5.464593	6.860355	6.779927	6.733288
beta-1,3-N-acetylgalactosaminyltransferase 1 (globoside blood group)	5.252577	5.33131	5.351121	6.488488	6.4146	6.295964
insulin-like growth factor 2 receptor	8.640657	8.510571	8.550467	9.608947	9.717721	9.640278
ATP-binding cassette, sub-family C (CFTR/MRP), member 3	5.554767	5.737082	5.884078	6.838624	6.794308	6.808413
---	4.105025	4.205007	4.115631	5.133105	5.31797	5.246871
glycine receptor, beta	4.750069	4.509476	4.625577	5.830582	5.773119	5.554642
CDNA clone IMAGE:3927515	5.936808	6.044012	6.189058	7.309696	7.177282	6.960963
ATP/GTP binding protein-like 5	4.784093	4.581689	4.775205	5.885456	5.538033	6.009319
Ras association (RalGDS/AF-6) domain family 8	5.653386	5.789154	5.961059	6.819744	6.914233	6.964093
neural precursor cell expressed, developmentally down-regulated 9	3.890436	3.441589	3.327151	4.542134	4.698595	4.716417
collagen, type XII, alpha 1	4.070984	4.190164	3.705485	4.853496	5.226109	5.186342
AXL receptor tyrosine kinase	9.182719	9.163906	9.369661	10.38383	10.30367	10.32866
coactosin-like 1 (Dictyostelium)	11.14915	10.93848	11.19687	12.23368	12.16357	12.1902
integrin, beta-like 1 (with EGF-like repeat domains)	4.634967	4.588726	4.918188	6.170836	5.558608	5.734262
LAG1 homolog, ceramide synthase 2	10.49199	10.29704	10.45813	11.60906	11.46794	11.49678
nephronectin	5.007698	5.410534	5.102757	6.415576	6.407958	6.026828
SH2 domain containing 5	6.347715	6.233683	6.299243	7.396698	7.333626	7.481454
ELOVL family member 7, elongation of long chain fatty acids (yeast)	7.334808	7.248761	7.375435	8.434327	8.439444	8.422805
tubulin, beta 2B	3.68661	3.680273	3.685987	5.062321	4.562034	4.769399
Homo sapiens, clone IMAGE:4393354, mRNA	3.270748	3.467787	3.241934	4.474034	4.599129	4.250821
jun oncogene	6.339174	6.31865	6.269483	7.471136	7.432123	7.370078
leukemia inhibitory factor receptor alpha	4.73222	5.079692	4.884237	5.950741	6.161906	5.931227
lymphotoxin beta (TNF superfamily, member 3)	6.122545	6.120233	6.157406	7.145622	7.190004	7.415321
heterogeneous nuclear ribonucleoprotein U	6.726603	6.636645	6.569886	7.66007	8.008574	7.616323
hypothetical gene supported by AK091454	3.153672	3.368815	3.24433	4.435202	4.454514	4.232889
Transcribed locus	5.694742	5.385698	5.16082	6.600659	6.553134	6.445197
TNFAIP3 interacting protein 1	9.028851	9.220739	9.18936	10.33987	10.26543	10.19441
polymerase (RNA) III (DNA directed) polypeptide C (62kD)	7.847532	7.759233	8.006146	8.984135	9.114622	8.877126
CDNA clone IMAGE:4819084	6.146888	6.307428	6.0189	7.343257	7.300453	7.194118
CDNA FLJ40762 fis, clone TRACH2002847	8.032282	7.977363	7.822631	9.100666	9.091134	9.008039

family with sequence similarity 43, member A	6.009999	6.091547	5.945309	7.185188	7.040312	7.190372
RAB38, member RAS oncogene family	9.338436	9.514332	9.404126	10.48524	10.61293	10.53231
ATPase, H+ transporting, lysosomal V0 subunit a4	3.713516	3.471036	3.648269	4.799611	4.764824	4.646711
glucosidase, beta (bile acid) 2	6.193753	5.975946	6.162729	7.130469	7.260607	7.324288
bone marrow stromal cell antigen 2	4.987058	4.865356	5.339424	6.192091	6.454352	5.935829
SMAD specific E3 ubiquitin protein ligase 2	6.007629	5.956428	6.052938	7.310616	7.120002	6.976875
interleukin 7 receptor	6.638208	6.458625	6.549794	7.768589	7.504488	7.767438
STAM binding protein-like 1	7.522994	7.324056	7.617296	8.550877	8.669514	8.6384
lipase, endothelial	5.189956	4.847304	5.349589	6.081388	6.559886	6.143373
family with sequence similarity 107, member B	4.716376	4.526338	3.954875	5.568942	5.748808	5.285192
transmembrane and tetratricopeptide repeat containing 2	4.848083	4.580285	4.76674	6.010493	5.756893	5.840878
hepatocyte nuclear factor 4, gamma	3.703278	3.991294	3.606603	4.793026	5.192871	4.735876
Transcribed locus, moderately similar to XP_948623.1	3.536065	3.305445	3.608022	4.780814	4.257823	4.83678
delta-like 1 (Drosophila)	7.418823	7.174786	7.565119	8.654676	8.568738	8.362631
dual adaptor of phosphotyrosine and 3-phosphoinositides	5.026913	5.273012	4.957588	6.034605	6.272063	6.379586
G protein-coupled receptor 39	5.125025	4.971831	5.512469	6.530951	6.361304	6.151653
deleted in liver cancer 1	7.10233	6.631213	6.937492	8.089509	7.899646	8.119782
hypothetical protein FLJ32549	5.185537	5.046128	5.072715	6.493308	5.910459	6.3478
follistatin	6.323808	5.803576	6.201828	7.308802	7.274538	7.208681
N-acetylneuraminic acid synthase (sialic acid synthase)	10.54865	10.40431	10.5503	11.71807	11.61425	11.64408
Rho GTPase activating protein 29	5.872572	5.426445	6.00547	6.978193	6.689803	7.109945
TBC1 domain family, member 9 (with GRAM domain)	5.903148	5.654872	5.670006	6.855005	6.973585	6.874075
glycine dehydrogenase (decarboxylating)	4.802586	4.993222	5.142556	6.003129	6.023112	6.388925
adaptor-related protein complex 1, sigma 3 subunit	4.918626	4.797299	4.384425	5.923813	5.718609	5.936592
zinc finger protein 556	5.033897	5.227992	5.439689	6.323586	6.132388	6.727567
glutamate-cysteine ligase, modifier subunit	6.571486	6.464164	6.383511	8.000696	7.282056	7.619404
integrin, beta 4	9.557122	9.247112	9.77854	10.7029	10.66357	10.70341
tumor necrosis factor, alpha-induced protein 3	6.562594	6.419792	6.681857	7.724489	7.608284	7.821652
transglutaminase 2	5.684599	5.487167	5.668745	6.805216	6.565921	6.962525
aspartate beta-hydroxylase domain containing 1	5.085054	5.313447	5.254462	6.719489	6.272063	6.156322
placenta-specific 8	7.616925	7.454743	7.352692	8.798376	8.683437	8.437624
similar to Arylacetamide deacetylase (AADAC)	3.2377	3.258407	3.250663	4.345688	4.842405	4.061661
Interleukin 6 signal transducer (gp130, oncostatin M receptor)	9.154228	8.962957	9.022507	10.16049	10.13393	10.34912
tumor necrosis factor, alpha-induced protein 3	7.692682	7.619345	7.769347	8.825531	8.83917	8.938752

intercellular adhesion molecule 1 (CD54)	7.393973	7.494206	7.443577	8.612389	8.681864	8.569241
PDZ and LIM domain 2 (mystique)	6.767674	6.779866	6.972763	8.217895	7.939163	7.898416
translocation associated membrane protein 2	6.314749	5.998721	6.224597	7.536623	7.022888	7.514547
zinc finger protein 697	5.546772	5.384715	5.457498	6.446141	6.850748	6.630287
B-cell receptor-associated protein 29	4.051918	3.79206	4.06442	5.314181	5.083436	5.049889
eukaryotic translation initiation factor 5A2	6.732199	6.724341	6.891648	7.926054	8.036631	7.925691
fibroblast activation protein, alpha	4.476968	4.631546	4.375696	5.624286	5.695025	5.707472
ADAM metallopeptidase with thrombospondin type 1 motif, 3	2.874774	2.967596	2.932402	3.905416	4.392292	4.0218
Transcribed locus	3.68471	3.381996	3.692799	4.983083	4.782201	4.539309
vitamin D (1,25- dihydroxyvitamin D3) receptor	6.225939	6.231586	6.290267	7.492713	7.386316	7.417089
CDNA FLJ31398 fis, clone NT2NE1000175	5.653165	5.532382	6.050154	6.982194	7.08089	6.724761
nuclear receptor subfamily 2, group E, member 1	3.671831	3.749008	3.82424	4.775414	5.199739	4.822848
Transcribed locus	6.219362	5.621781	6.061765	7.185646	7.154044	7.119634
phosphoinositide-3-kinase, regulatory subunit 3 (p55, gamma)	6.417994	6.28792	6.439826	7.568895	7.560869	7.576831
NEL-like 2 (chicken)	4.51649	4.326911	4.250532	5.829115	5.595363	5.239875
deleted in liver cancer 1	7.820807	7.52185	7.990491	9.062143	8.899776	8.948206
ISL LIM homeobox 1	5.652548	5.567222	5.171412	6.542847	6.721416	6.705817
plasminogen activator, urokinase receptor	6.693649	6.484361	6.506237	7.772789	7.773026	7.722066
E2F transcription factor 7	7.953949	7.872645	8.021966	9.127768	9.268364	9.045294
osteopetrosis associated transmembrane protein 1	7.152135	7.08831	7.197163	8.292131	8.427668	8.312939
activating transcription factor 3	5.685039	6.330933	5.791556	7.390132	7.122919	6.890662
phytoceramidase, alkaline	6.82042	6.790148	6.813938	7.983863	8.020685	8.022396
Transcribed locus	4.864059	4.601282	4.701119	6.135299	5.980625	5.666038
Transcribed locus	6.33498	6.071103	5.783495	7.372062	7.262276	7.17649
islet cell autoantigen 1, 69kDa	5.270279	5.088134	4.95211	6.312329	6.212217	6.413588
hypothetical LOC401022	5.368515	5.054281	5.42595	6.612564	6.496534	6.376721
aldo-keto reductase family 1, member C2	5.550599	5.470339	5.551615	6.692101	6.74885	6.768888
---	5.891601	6.065466	5.984302	7.200874	7.336466	7.041533
Transcribed locus	7.042872	7.283186	7.029943	8.39976	8.308434	8.287203
suppressor of cytokine signaling 2	6.26501	6.001954	6.241613	7.452503	7.418574	7.287947
ELAV (embryonic lethal, abnormal vision, Drosophila)-like 2	6.34634	6.221636	6.421052	7.471933	7.725072	7.447001
Transcribed locus	6.406074	6.52094	6.474946	7.548648	7.765876	7.742891
nuclear receptor subfamily 1, group D, member 2	6.526152	6.096002	6.127859	7.424621	7.493356	7.489424
glycoprotein M6A	3.785769	4.099339	3.845783	5.293455	5.400122	4.695148

oxysterol binding protein-like 6	5.016372	4.924135	4.697209	6.261003	6.228259	5.806577
cAMP responsive element binding protein 3-like 2	7.498753	7.523075	7.410452	8.624658	8.757766	8.724629
thiamin pyrophosphokinase 1	3.358019	3.775734	3.820257	4.933524	5.114337	4.596275
osteopetrosis associated transmembrane protein 1	4.99125	5.203295	4.964207	6.305287	6.154022	6.39088
popeye domain containing 3	6.31195	6.102687	6.442089	7.528999	7.515184	7.506951
keratin 6B	4.765274	4.523683	5.014739	5.970087	6.307617	5.730447
glutamate-cysteine ligase, modifier subunit	6.193752	6.264809	6.202966	7.580141	7.334538	7.455837
microphthalmia-associated transcription factor	6.045452	5.63823	5.753785	7.021203	7.19654	6.928963
Similar to RIKEN cDNA 2310002J15 gene	6.718356	6.644936	6.682532	7.947736	7.998251	7.844733
dynein, cytoplasmic 1, intermediate chain 1	4.096391	3.873923	3.759484	5.399887	5.224024	4.853983
cathepsin O	3.832507	4.246498	3.932677	5.269905	5.334441	5.166183
5'-nucleotidase, ecto (CD73)	7.70117	7.365131	7.747838	8.857733	8.746752	8.970228
ADP-ribosylation factor-like 4C	7.664834	7.539806	7.521365	8.820704	8.77607	8.896863
fasciculation and elongation protein zeta 1 (zygin I)	9.168063	9.260571	9.30583	10.58079	10.43501	10.49354
protocadherin beta 2	6.077067	6.037486	5.985003	7.42753	7.126231	7.321972
CDNA FLJ13601 fis, clone PLACE1010069	2.965244	2.793587	2.753246	4.366483	4.111247	3.815546
choroideremia (Rab escort protein 1)	3.012088	2.741977	2.843462	3.801164	4.189117	4.390544
islet cell autoantigen 1, 69kDa	5.895344	6.017746	5.972779	7.118269	7.346308	7.206259
coactosin-like 1 (Dictyostelium)	10.21878	10.02075	10.22155	11.49621	11.35184	11.40406
oxysterol binding protein-like 6	6.214136	6.00406	6.151528	7.396702	7.364365	7.403195
MHC class I polypeptide-related sequence B	8.698164	8.425091	8.807365	10.01101	9.841255	9.873483
CDNA clone IMAGE:4837650	3.303869	3.216513	3.08482	4.290824	4.437756	4.675745
CDNA clone IMAGE:3840062	3.405672	3.666488	3.752747	4.773311	4.927068	4.924453
solute carrier family 22 (organic cation transporter), member 4	5.901075	5.675788	6.078908	7.140882	7.216527	7.103389
SATB homeobox 2	4.788995	5.154385	5.278641	6.230872	6.310103	6.488737
receptor accessory protein 1	6.502344	6.367243	6.478456	7.725567	7.859342	7.581017
methionine sulfoxide reductase B3	7.528596	7.319524	7.525932	8.878851	8.61675	8.699804
zinc finger protein 533	2.917876	3.025222	2.882247	4.039974	4.238462	4.369272
interleukin 6 (interferon, beta 2)	5.417828	5.230986	5.677075	6.739826	6.760137	6.651674
smoothelin	7.88885	7.612328	8.195333	9.177862	9.200555	9.145482
RNA pseudouridylate synthase domain containing 3	4.664514	4.10646	4.523892	5.813478	5.432795	5.883298
solute carrier family 4, sodium borate transporter, member 11	6.289807	6.095386	6.515352	7.688452	7.553916	7.496679
CDNA: FLJ23131 fis, clone LNG08502	3.176221	3.291722	2.98108	4.438777	4.484906	4.377548
Transcribed locus	4.964367	5.03383	4.996824	6.201125	6.455962	6.196665

cysteine-rich, angiogenic inducer, 61	9.611164	9.130436	9.667583	10.77974	10.67256	10.81957
Full-length cDNA clone CS0DF032YA11 of Fetal brain of Homo sapiens	4.146118	3.922477	3.966836	5.223871	5.11694	5.563354
pleiomorphic adenoma gene-like 1	5.800379	5.816492	5.654449	7.369537	6.633234	7.139238
chromosome 2 open reading frame 32	4.52564	4.700608	4.542318	5.946426	5.916394	5.779005
pleckstrin homology-like domain, family A, member 1	7.735551	7.689784	7.808767	8.972692	9.172756	8.97077
poliovirus receptor-related 3	5.615329	5.42991	5.380513	6.750296	6.776044	6.78471
leukemia inhibitory factor (cholinergic differentiation factor)	6.702516	6.536427	6.973506	8.083472	7.947515	8.070665
phytoceramidase, alkaline	7.874786	7.761729	7.677107	9.138095	8.937631	9.12962
Full-length cDNA clone CS0DD001YA12	4.588259	4.321186	4.489659	5.925571	5.790522	5.578677
dapper, antagonist of beta-catenin, homolog 2 (Xenopus laevis)	5.545852	5.544276	5.569036	6.962396	6.825532	6.77103
uridine phosphorylase 1	9.721186	9.633758	9.991259	11.07007	11.12671	11.04925
histone deacetylase 9	6.382527	6.180141	6.331697	7.673153	7.569018	7.554546
Ras-related GTP binding D	3.690418	3.758922	3.320834	5.05716	5.191835	4.43166
aspartate beta-hydroxylase domain containing 1	6.948982	6.9495	7.020249	8.356095	8.210588	8.271647
colony stimulating factor 3 (granulocyte)	6.206567	6.240026	6.374139	7.592723	7.405982	7.741772
smoothelin	8.449226	8.286766	8.676216	9.730318	9.787568	9.816281
SRY (sex determining region Y)-box 2	4.072008	3.839097	3.771397	5.23805	5.167009	5.202386
dedicator of cytokinesis 4	3.271151	3.663906	3.23805	4.699181	4.798319	4.612295
---	4.426584	4.599109	4.221634	5.668566	5.807088	5.714578
DnaJ (Hsp40) homolog, subfamily C, member 6	7.642134	7.1866	7.533647	8.891615	8.675845	8.740653
Surfactant associated protein F mRNA, partial sequence	5.588124	5.352163	5.520011	6.826982	6.764363	6.827042
uncoupling protein 2 (mitochondrial, proton carrier)	6.862689	7.079242	7.042909	8.630307	8.302205	8.026123
---	8.776319	8.769208	8.721304	10.04059	10.09226	10.10912
fibulin 1	7.597703	7.734642	7.89942	9.070653	9.084826	9.05362
serpin peptidase inhibitor, clade F	7.348403	7.198132	7.30657	8.714876	8.619328	8.517445
annexin A6	5.724067	5.521398	5.940363	7.055428	7.032268	7.098743
ventricular zone expressed PH domain homolog 1 (zebrafish)	4.550474	4.793409	4.366016	5.977133	5.999389	5.73825
MRNA (clone ICRFp507I1077)	6.202072	6.201801	6.175664	7.45065	7.537946	7.596604
RGM domain family, member B	6.187674	6.109055	6.166656	7.563668	7.291101	7.616005
transmembrane, prostate androgen induced RNA	5.977306	5.271875	6.380518	7.189826	7.261257	7.190309
methionine sulfoxide reductase B3	7.720604	7.561754	7.633792	9.033078	8.942306	8.971315
pleckstrin homology-like domain, family A, member 1	9.865619	9.50636	9.751635	11.12384	11.061	10.9743
secreted protein, acidic, cysteine-rich (osteonectin)	4.347175	4.482851	4.650951	5.968082	5.778017	5.772422
protein kinase, cAMP-dependent, regulatory, type II, beta	4.449142	4.539311	4.582811	6.287495	5.785779	5.53739

transmembrane protein with EGF-like and two follistatin-like domains 1	5.108508	4.748429	5.047149	6.129735	6.383454	6.438626
phospholipid scramblase 4	3.199229	3.518375	3.399225	4.631563	4.834132	4.699363
NLR family, pyrin domain containing 1	4.776917	5.459535	4.888031	6.491763	6.416831	6.266929
ALX homeobox 1	4.414836	4.511932	4.342496	5.919482	5.521029	5.880523
CAP-GLY domain containing linker protein 2	5.765908	5.869754	6.264761	7.279059	7.447717	7.225703
protein kinase (cAMP-dependent, catalytic) inhibitor beta	3.888493	4.136792	3.600997	5.269906	5.584235	4.842424
family with sequence similarity 134, member B	3.895072	4.059716	4.013691	5.303008	5.560999	5.179609
neuron navigator 3	5.549294	4.927825	5.107176	6.176406	6.813921	6.671401
pleckstrin homology-like domain, family A, member 1	5.967021	5.645573	5.882795	7.004594	7.340269	7.230308
RAB3B, member RAS oncogene family	6.793984	6.472492	6.787149	7.886764	8.440568	7.816175
family with sequence similarity 107, member B	3.771567	3.76104	4.030961	5.288861	5.222266	5.143779
glucosamine (UDP-N-acetyl)-2-epimerase/N-ace. kinase	4.009609	4.542494	3.812165	5.499121	5.524881	5.449539
cysteine-rich, angiogenic inducer, 61	8.473805	8.01845	8.601141	9.655242	9.672213	9.881342
Transcribed locus	4.004795	4.26468	4.030961	5.949221	5.609807	4.867412
ring finger protein 182	4.368105	3.906135	4.05676	5.487587	5.57435	5.401605
heparin-binding EGF-like growth factor	7.174553	7.078938	7.226088	8.608818	8.555548	8.455372
ornithine decarboxylase 1	10.771	10.70709	10.98021	12.0851	12.27768	12.24297
fibroblast growth factor 2 (basic)	6.868116	6.863002	6.917864	8.341422	8.373128	8.08659
solute carrier family 6, member 15	3.972361	3.953818	3.61844	5.298464	5.500448	4.91927
interleukin 1 receptor, type II	3.866957	3.754773	3.338621	5.161125	4.855473	5.119282
Transcribed locus	6.298443	6.26439	6.009909	7.601994	7.645436	7.52266
engulfment and cell motility 1	4.78357	4.83864	4.732172	6.437732	6.047711	6.103908
integrin, alpha 5 (fibronectin receptor, alpha polypeptide)	6.566936	6.542519	6.798291	8.061311	8.106884	7.97864
solute carrier family 45, member 3	6.506476	6.525848	6.775791	8.216236	7.923051	7.921702
Unknown mRNA sequence	4.386535	4.215105	4.250179	5.716722	5.681176	5.708255
hippocalcin-like 1	9.078809	8.919837	9.086002	10.49039	10.38313	10.47224
interleukin 1, beta	9.458657	9.436919	9.328459	10.9148	10.85782	10.71772
serine incorporator 2	8.103514	8.074423	8.233902	9.650736	9.504425	9.532539
sterile alpha motif domain containing 5	5.103526	4.450239	5.017069	6.164782	6.420939	6.264008
pleiomorphic adenoma gene-like 1	6.443401	6.138597	6.135389	7.817285	7.628866	7.560855
solute carrier family 20 (phosphate transporter), member 1	10.55056	10.43794	10.66657	11.94404	12.03518	11.97343
hypothetical gene supported by BC013438	6.214136	5.675334	5.89913	7.567398	7.234985	7.312226
ankyrin repeat domain 6	5.139435	5.285437	5.294801	6.679041	6.669468	6.701603
transglutaminase 2	7.381998	7.159148	7.484912	8.988897	8.607292	8.764879

---	4.83164	4.192935	5.091423	5.863142	6.259767	6.333658
NF of kappa light polypeptide gene enhancer in B-cells inhibitor, zeta	7.322468	7.104309	7.005343	8.536018	8.599294	8.637693
RAB32, member RAS oncogene family	9.143021	9.095448	9.328234	10.7364	10.53817	10.64161
Keratin 7	3.976345	3.876619	4.02328	4.75184	5.618214	5.862011
mucolipin 2	5.786814	5.689666	5.609376	7.070777	7.217831	7.157011
hypothetical protein LOC643837	5.536105	5.285504	5.262284	6.864305	6.821411	6.758657
LIM and calponin homology domains 1	4.365801	4.442939	3.816991	5.614212	5.565079	5.809425
beta-1,3-N-acetylgalactosaminyltransferase 1	5.616748	5.402495	5.602669	6.963002	7.049031	7.002564
transmembrane protein with EGF-like and two follistatin-like domains 1	5.974057	5.83545	5.523279	7.460893	6.947521	7.330908
SRY (sex determining region Y)-box 9	6.382612	6.083526	6.432047	7.89201	7.521922	7.893265
pleiomorphic adenoma gene-like 1	6.622463	6.699647	6.507727	8.195602	8.000204	8.048621
CDNA FLJ43100 fis, clone CTONG2003100	3.74701	3.495829	3.74473	5.014662	5.334571	5.056183
heparin-binding EGF-like growth factor	6.390326	6.250643	6.623405	7.728009	7.898374	8.058993
neuropilin 1	3.025435	3.243569	3.377932	4.767595	4.955172	4.348042
Schlafen family member 5	4.019034	3.967234	3.998772	5.181433	5.966263	5.268264
transmembrane protein 5	6.585868	6.388759	6.441434	8.067813	7.893115	7.896025
chemokine (C-X-C motif) ligand 2	8.256428	7.92687	8.180986	9.647849	9.599262	9.589507
A kinase (PRKA) anchor protein (gravin) 12	6.487978	6.270553	6.583874	7.883277	7.930993	8.002421
PDZK1 interacting protein 1	4.590167	4.911477	5.11433	6.58677	6.152124	6.372298
leucine-rich alpha-2-glycoprotein 1	5.390415	5.418963	5.199744	7.249093	6.837912	6.441872
phytoceramidase, alkaline	7.698272	7.493901	7.370057	9.005584	9.066348	9.01649
V-set and immunoglobulin domain containing 1	3.60598	3.817753	3.752628	5.349912	5.31662	5.039583
serum amyloid A1	6.037517	5.738672	5.335358	7.262634	7.305326	7.08929
connective tissue growth factor	8.554316	8.167832	8.931562	9.969222	10.07508	10.15988
beta-1,3-N-acetylgalactosaminyltransferase 1	4.544642	4.624497	4.76664	6.263669	6.024817	6.209003
coronin, actin binding protein, 2A	7.363164	7.565577	7.380935	9.092901	8.947245	8.837252
interleukin 1, beta	9.729236	9.703632	9.757474	11.24172	11.37388	11.17366
colony stimulating factor 2 receptor, alpha, low-affinity	5.033822	5.063329	5.120087	6.46768	6.886812	6.499963
Golgi SNAP receptor complex member 2	3.808066	3.657394	3.83866	5.393604	5.446978	5.101517
LIM and calponin homology domains 1	4.575704	4.568781	4.27685	6.103702	5.862105	6.094265
5'-nucleotidase, ecto (CD73)	8.538082	8.565601	8.577314	10.04915	10.1055	10.19145
anthrax toxin receptor 2	6.454694	6.089777	6.028903	7.60847	7.858392	7.790765
transmembrane protein 2	4.953207	4.947987	5.127544	6.394545	6.655715	6.665293
diacylglycerol O-acyltransferase homolog 2 (mouse)	5.445921	5.882092	5.126744	7.015409	6.967801	7.170617

fucosyltransferase 4 (alpha (1,3) fucosyltransferase, myeloid-specific)	4.315771	4.23137	4.388901	5.995248	5.874394	5.76667
---	5.060579	5.051709	5.212978	6.481581	6.854099	6.69299
monooxygenase, DBH-like 1	4.564985	4.386003	4.675704	6.102021	6.379987	5.852736
Sp5 transcription factor	5.833477	5.833922	5.985548	7.387283	7.325245	7.650785
ADP-ribosylation factor-like 4C	7.575144	7.39585	7.639239	9.049631	9.19946	9.071836
adrenomedullin	9.794618	10.14297	9.546629	11.39014	11.3328	11.52611
RNA binding motif, single stranded interacting protein	4.181672	4.109375	4.24311	5.965117	5.721202	5.619978
lung cancer metastasis-associated protein	2.852862	3.030511	2.731554	4.45233	4.546466	4.391688
intercellular adhesion molecule 1 (CD54), human rhinovirus receptor	6.162444	6.052951	6.227436	7.681315	7.899882	7.670587
protocadherin 7	5.689666	5.682086	6.322709	7.549895	7.450169	7.512603
SRY (sex determining region Y)-box 9	6.945812	6.798883	7.025412	8.468758	8.417603	8.70747
ornithine decarboxylase antizyme 3	5.700572	5.540909	5.638779	7.31541	7.16553	7.23671
regulator of G-protein signaling 2, 24kDa	6.104695	6.038985	5.979278	7.558168	7.797222	7.614309
linker for activation of T cells family, member 2	5.012503	4.899307	5.103178	6.800477	6.391539	6.678784
asparaginase like 1	6.154796	5.925554	6.145998	7.805937	7.762787	7.523031
A kinase (PRKA) anchor protein (gravin) 12	8.516876	8.42469	8.740395	10.06667	10.30549	10.18932
interleukin 7 receptor	6.286878	5.997424	6.626173	7.847581	7.978399	7.988467
protocadherin beta 16	3.09526	3.027737	3.008193	4.80272	4.698595	4.559847
3'-phosphoadenosine 5'-phosphosulfate synthase 2	5.746624	5.618519	5.727412	7.341773	7.31128	7.42274
protocadherin 7	7.3838	6.962646	7.333074	8.886827	8.923656	8.854695
fibulin 1	6.886918	6.963861	7.021066	8.635213	8.637318	8.598816
progressive rod-cone degeneration	5.188979	5.399661	5.487375	7.13972	7.133885	6.816985
CUG triplet repeat, RNA binding protein 2	3.654435	3.563448	3.342858	5.085302	4.838505	5.675015
lysosomal-associated membrane protein 3	7.642258	7.631174	7.464104	9.35713	9.253246	9.171935
coronin, actin binding protein, 2B	5.319315	5.250637	5.683304	7.183189	7.018819	7.128093
hippocalcin-like 1	7.292087	7.07536	7.306164	9.004875	8.78512	8.964612
solute carrier family 1	4.23989	4.501546	4.224813	6.082637	6.164974	5.809545
G protein-coupled receptor 37 (endothelin receptor type B-like)	2.982248	3.092746	2.915108	4.878059	4.69385	4.529966
collagen, type XII, alpha 1	5.023295	4.730117	4.895977	6.555543	6.696	6.513034
ribosomal protein S6 kinase, 90kDa, polypeptide 2	3.645951	4.047909	4.522895	5.879426	5.962925	5.509512
pleckstrin homology-like domain, family A, member 1	7.948432	7.747171	7.916194	9.601821	9.608662	9.570598
guanylate cyclase 1, soluble, beta 3	3.237363	3.17537	3.419255	4.743111	5.304184	4.954715
brain-derived neurotrophic factor	5.616573	5.387323	5.19505	7.098324	7.271834	7.006173
kynureninase (L-kynurenine hydrolase)	2.82453	2.746273	3.063693	4.11459	5.03304	4.667787

ADP-ribosylation factor-like 4C	8.213484	8.068655	8.081749	9.855722	9.841513	9.856702
PDZK1 interacting protein 1	4.795151	4.450239	4.452482	6.22089	6.383895	6.288231
cytoglobin	5.835039	6.078557	6.287827	7.913237	7.859345	7.64241
protein kinase C, alpha	5.305558	4.81538	4.847378	6.71602	6.873415	6.606566
interleukin 1, alpha	9.95745	9.867037	9.853376	11.58167	11.60733	11.74106
UDP-N-acetyl-alpha-GalNAc-T6	6.4117	6.154924	6.442089	8.192796	8.021918	8.058777
protein kinase (cAMP-dependent, catalytic) inhibitor alpha	5.753823	5.831019	5.809387	7.591147	7.516617	7.552555
serum amyloid A1 /// serum amyloid A2	4.563557	4.899022	4.101831	6.461419	6.341896	6.027482
SEC14 and spectrin domains 1	4.784093	4.197962	4.599558	6.287766	6.467802	6.093089
cell adhesion molecule 1	6.246067	6.158702	6.513572	8.157269	8.024227	8.03403
interleukin 8	9.081554	8.888514	9.085896	10.74885	10.71241	10.93449
G protein-coupled receptor 63	4.077567	4.220072	4.273403	5.885804	6.025906	6.001893
mucolipin 2	5.043113	5.297588	5.111223	6.790574	7.023518	7.011815
Transcribed locus	7.976418	7.867789	7.861955	9.675045	9.756836	9.684877
aldo-keto reductase family 1, member C3	3.937533	3.60924	3.774405	5.64909	5.578135	5.511886
fibulin 1	6.317185	6.34466	6.483198	8.248633	8.160756	8.155383
coiled-coil domain containing 46	3.848004	3.570186	3.744729	5.315242	6.157136	5.121652
protein phosphatase 1K (PP2C domain containing)	5.831373	5.467973	5.800683	7.601697	7.439168	7.517741
endoglin (Osler-Rendu-Weber syndrome 1)	5.982009	6.133496	6.356635	8.173852	7.988946	7.818208
ecotropic viral integration site 1	5.832434	6.381627	6.020714	7.780751	8.126251	7.868752
3'-phosphoadenosine 5'-phosphosulfate synthase 2	7.120996	6.938441	7.186255	9.047319	8.914534	8.858727
peptidyl arginine deiminase, type III	6.11145	6.307693	6.169251	8.435841	7.678926	8.074047
tetraspanin 12	4.233586	4.291019	4.06429	6.002865	6.140812	6.057811
collagen, type VIII, alpha 1	2.823671	2.706995	2.858521	4.879891	4.804218	4.352414
CUG triplet repeat, RNA binding protein 2	6.23425	6.676588	6.59621	8.309252	8.569195	8.277184
translocation associated membrane protein 2	8.582603	8.519625	8.54317	10.37241	10.40869	10.53032
transmembrane protein 40	6.124274	5.888322	6.070964	7.962989	7.813437	8.014241
dual adaptor of phosphotyrosine and 3-phosphoinositides	5.343811	4.485914	4.929987	6.808546	6.833022	6.836343
Protein kinase (cAMP-dependent, catalytic) inhibitor alpha	3.446663	3.512404	3.574134	5.385626	5.524027	5.353459
UDP-N-acetyl-alpha-GalNAc-T6	6.344172	6.497559	6.696437	8.56028	8.423965	8.408895
translocation associated membrane protein 2	7.574218	7.618979	7.804733	9.627021	9.658262	9.637953
CDNA clone IMAGE:4814828	5.561118	5.431669	5.4646	7.375489	7.660135	7.379473
integrin, beta-like 1 (with EGF-like repeat domains)	3.269432	3.174851	3.249575	5.124881	5.828214	4.725124
protocadherin 7	6.087585	5.866523	6.228959	7.932737	8.231157	8.021492

monooxygenase, DBH-like 1	3.900604	3.956194	4.132072	5.870772	6.400627	5.735006
brain-derived neurotrophic factor	4.472197	4.679328	4.924339	6.818868	6.648932	6.659558
interleukin 8	7.83098	7.581461	7.815917	9.464786	9.777572	10.04437
thiamin pyrophosphokinase 1	3.689322	4.083318	3.889933	5.981501	6.320949	5.462033
endothelin 1	5.884665	5.500223	6.025326	7.790292	7.861346	7.935318
integrin, beta-like 1 (with EGF-like repeat domains)	3.616074	3.560086	3.547743	6.492524	5.222267	5.203326
fibroblast growth factor 2 (basic)	4.469465	4.153702	4.10886	6.472593	6.100047	6.358146
endothelial differentiation, sphingolipid G-protein-coupled receptor, 3	4.452956	4.402712	4.535222	6.103985	6.699785	6.823241
ecotropic viral integration site 1	5.084945	4.763571	5.065624	7.026982	7.27544	6.870478
dual specificity phosphatase 6	6.746661	6.807915	7.011415	8.942148	8.950434	8.963319
LIM and calponin homology domains 1	4.082133	3.685785	3.747983	5.955695	5.864752	6.014961
transmembrane protein 40	7.945268	7.996919	8.213926	10.20282	10.12611	10.20111
Cystatin E/M	5.189143	4.886703	5.614621	7.435096	7.41001	7.297683
CDNA FLJ30478 fis, clone BRAWH1000167	3.345866	3.436826	3.085536	5.421644	5.438098	5.547002
guanine nucleotide binding protein (G protein), gamma 11	4.799589	4.822149	4.895977	6.931329	7.176698	7.000362
Transcribed locus	4.210316	4.748376	4.367378	6.84519	6.587094	6.575099
similar to Complement C3 precursor	8.888649	8.935467	8.741045	11.06983	11.1762	11.03963
Contactin 1	4.071926	4.101915	3.634004	6.073518	6.295592	6.159392
glycoprotein M6A	3.357057	3.140793	3.5986	5.69379	5.416804	5.707017
quaking homolog, KH domain RNA binding (mouse)	4.721305	4.259231	4.306058	6.666835	6.749846	6.685325
cytochrome P450, family 27, subfamily B, polypeptide 1	7.360508	6.730802	7.495443	9.542182	9.470119	9.518998
chemokine (C-X-C motif) ligand 1	9.09614	8.870655	9.058304	11.52138	11.10993	11.35228
endothelin 1	4.695026	4.567499	5.145396	7.246648	7.064434	7.117097
copine VIII	3.175336	3.286235	3.632607	5.640396	5.886122	5.595353
collagen, type XII, alpha 1	4.97808	4.665255	5.311276	7.336573	7.394521	7.262517
suppressor of cytokine signaling 3	3.921787	3.819388	4.029675	6.655787	6.019207	6.289073
prostaglandin E receptor 4 (subtype EP4)	3.923803	3.715308	3.889933	6.264759	6.279616	6.184841
dual specificity phosphatase 6	7.283442	6.947522	7.375688	9.856815	9.398129	9.659339
pentraxin-related gene, rapidly induced by IL-1 beta	4.082132	3.975914	3.796556	6.593229	6.125235	6.461476
dual specificity phosphatase 6	6.654125	6.286632	6.75559	9.192919	8.795338	9.141846
CDNA FLJ13598 fis, clone PLACE1009921	5.067607	4.621228	5.009006	7.49539	7.574385	7.202101
WNT1 inducible signaling pathway protein 3	4.989868	5.14292	5.643754	7.813363	7.8722	7.701625
serpin peptidase inhibitor, clade I (neuroserpin), member 1	4.228648	4.592157	4.487325	7.033589	7.127471	6.767828
cell adhesion molecule 1	4.994322	4.884007	5.261337	7.455748	7.646064	7.701262

endothelin 1	6.006515	5.800423	6.512631	8.861431	8.709753	8.965166
solute carrier family 2 (facilitated glucose transporter), member 13	3.906086	3.677135	3.625964	6.503545	6.423594	6.554756
microfibrillar-associated protein 3-like	3.191432	3.033106	3.096482	5.603862	6.190824	5.819984
A kinase (PRKA) anchor protein 2 /// PALM2-AKAP2 protein	4.269479	4.610632	4.255682	7.169746	7.293475	7.111384
roundabout, axon guidance receptor, homolog 1 (Drosophila)	3.903023	4.326911	4.335662	7.08049	7.171082	6.790061
interleukin 32	6.199085	6.340094	6.513164	9.289092	9.129719	9.184414
transmembrane protein 133	3.244177	3.392783	3.059059	6.244664	6.151382	5.943846
vimentin	5.080086	5.288553	5.841584	8.342526	8.427337	8.363849
A kinase (PRKA) anchor protein 2 /// PALM2-AKAP2 protein	3.957505	4.044418	3.601139	6.968772	6.862112	6.965442
lymphoid enhancer-binding factor 1	3.745839	3.611931	3.67239	6.794576	6.925491	6.852406
calmegin	3.266219	3.487106	3.618585	6.545059	6.80343	6.597862
A kinase (PRKA) anchor protein 2 /// PALM2-AKAP2 protein	4.607906	4.222429	4.307065	8.063871	8.122435	7.930062
similar to keratin associated protein 2-4	4.095901	4.102846	4.239194	7.848619	7.741798	8.148486
hypothetical protein FLJ32810	3.141547	3.331569	3.431438	7.16272	7.587752	7.131957
---	4.567354	4.454154	4.552277	8.758574	8.480986	8.654393
keratin 7	7.871861	7.835593	8.13014	12.42599	12.35343	12.24643
chemokine (C-C motif) ligand 20	4.756943	4.617249	4.902618	9.064451	9.249067	9.31394
MRNA full length insert cDNA clone EUROIMAGE 1913076	3.476054	3.376385	3.366153	8.330351	8.495708	8.409832
